# Thompson Sampling for Stochastic Bandits with Noisy Contexts: An Information-Theoretic Regret Analysis

**DOI:** 10.3390/e26070606

**Published:** 2024-07-17

**Authors:** Sharu Theresa Jose, Shana Moothedath

**Affiliations:** 1 School of Computer Science, University of Birmingham, Birmingham B15 2TT, UK; 2 Department of Electrical Engineering, Iowa State University, Ames, IA 50011, USA

**Keywords:** noisy contextual bandits, Thompson sampling, Bayes regret, information theory

## Abstract

We study stochastic linear contextual bandits (CB) where the agent observes a *noisy* version of the true context through a noise channel with unknown channel parameters. Our objective is to design an action policy that can “approximate” that of a Bayesian oracle that has access to the reward model and the noise channel parameter. We introduce a modified Thompson sampling algorithm and analyze its Bayesian cumulative regret with respect to the oracle action policy via information-theoretic tools. For Gaussian bandits with Gaussian context noise, our information-theoretic analysis shows that under certain conditions on the prior variance, the Bayesian cumulative regret scales as $\tilde{O}\left(m\sqrt{T}\right)$, where *m* is the dimension of the feature vector and *T* is the time horizon. We also consider the problem setting where the agent observes the true context with some delay after receiving the reward, and show that delayed true contexts lead to lower regret. Finally, we empirically demonstrate the performance of the proposed algorithms against baselines.

## 1. Introduction

Decision-making in the face of uncertainty is a widespread challenge found across various domains such as control and robotics [1], clinical trials [2], communications [3], and ecology [4]. To tackle this challenge, learning algorithms have been developed to uncover effective policies for optimal decision-making. One notable framework for addressing this is contextual bandits (CBs), which capture the essence of sequential decision-making by incorporating side information, termed *context* [5].

In the standard CB model, an agent interacts with the environment over numerous rounds. In each round, the environment presents a context to the agent based on which the agent chooses an action and receives a reward from the environment. The reward is stochastic, drawn from a probability distribution whose mean reward (which is a function of context-action pair) is unknown to the agent. The goal of the agent is to design a policy for action selection that can maximize the cumulative mean reward accrued over a *T*-length horizon.

In this paper, we focus on a CB model that assumes stochastic rewards with linear mean-reward functions, also called stochastic linear contextual bandits. Stochastic linear CB models find applications in various settings including internet advertisement selection [6], where the advertisement (i.e., action) and webpage features (i.e., context) are used to construct a linear predictor of the probability that a user clicks on a given advertisement, and article recommendation on web portals [7].

While most prior research on CBs has primarily focused on models with known exact contexts [8,9,10], in many real-world applications, the contexts are noisy, e.g., imprecise measurement of patient conditions in clinical trials, weather or stock market predictions. In such scenarios, when the exact contexts are unknown, the agent must utilize the observed noisy contexts to estimate the mean reward associated with the true context. However, this results in a biased estimate that renders the application of standard CB algorithms unsuitable. Consequently, recent efforts have been made to develop CB algorithms tailored to noisy context settings.

*Related Works*: ref. [11] considers a setting where there is a bounded zero-mean noise in the *m*-dimensional *feature vector* (denoted by ϕ(a,c), where *a* is the action and *c* is the context) rather than in the context vector, and the agent observes only noisy features. For this setting, they develop an upper confidence bound (UCB) algorithm. Ref. [12] models the uncertainty regarding the true contexts by a *context distribution* that is known to the agent, while the agent never observes the true context and develops a UCB algorithm. A similar setting has also been considered in [13]. Differing from these works, ref. [14] considers the setting where the true feature vectors are sampled from an unknown feature distribution at each time, but the agent observes only a noisy feature vector. Assuming Gaussian feature noise with unknown mean and covariance, they develop an Optimism in the Face of Uncertainty (OFUL) algorithm. A variant of this setting has been studied in [15].

*Motivation and Problem Setting:* In this work, inspired by [14], we consider the following noisy CB setting. In each round, the environment samples a true context vector $c_{t}$ from a *context distribution* that is *known* to the agent. The agent, however, does not observe the true context but observes a noisy context ${\hat{c}}_{t}$ obtained as the output of a noise channel $P({\hat{c}}_{t}|c_{t},\gamma^{*})$ parameterized by $\gamma^{*}$. The agent is aware of the noise present but does not know the channel parameter $\gamma^{*}$. Following [14], we consider Gaussian noise channels for our regret analysis.

Based on the observed noisy contexts, the agent chooses an action $a_{t}$ and observes a reward $r_{t}$ corresponding to the true context. We consider a linear bandit whose mean reward $\phi {\left(a_{t},c_{t}\right)}^{⊤}\theta^{*}$ is determined by an unknown reward parameter $\theta^{*}$. The goal of the agent is to design an action policy that minimizes the *Bayesian cumulative regret* with respect to the action policy of a Bayesian oracle. The oracle has access to the reward model and the channel parameter $\gamma^{*}$, and uses the predictive distribution of the true context given the observed noisy context to select an action.

Our setting differs from [14] in that we assume noisy contexts rather than noisy feature vectors and that the agent knows the context distribution. The noise model, incorporating noise in the feature vector, allows [14] to transform the original problem into a different CB problem that estimates a modified reward parameter. Such a transformation, however, is not straightforward in our setting with noise in contexts rather than in feature vectors, where we wish to analyze the Bayesian regret. Additionally, we propose a de-noising approach to estimate the predictive distribution of the true context from given noisy contexts, offering potential benefits for future analyses.

The assumption of known context distribution follows from [12]. This can be motivated by considering the example of an online recommendation engine that pre-processes the user account registration information or contexts (e.g., age, gender, device, location, item preferences) to group them into different clusters [16]. The engine can then infer the ‘empirical’ distribution of users within each cluster to define a context distribution over true contextual information. A noisy contextual information scenario occurs when a guest with different preferences logs into a user’s account.

*Challenges and Novelty:* Different from existing works that developed UCB-based algorithms, we propose a fully Bayesian Thompson Sampling (TS) algorithm that approximates the Bayesian oracle policy. The proposed algorithm differs from the standard contextual TS [10] in the following aspects. Firstly, since the true context vectors are not accessible at each round and the channel parameter $\gamma^{*}$ is unknown, the agent uses its knowledge of the context distribution and the past observed noisy contexts to infer a *predictive posterior* distribution of the true context from the current observed noisy context. The inferred predictive distribution is then used to choose the action. This *de-noising* step enables our algorithm to ‘approximate’ the oracle action policy that uses knowledge of the channel parameter $\gamma^{*}$ to implement *exact de-noising*. Secondly, the reward $r_{t}$ received by the agent corresponds to the unobserved true context $c_{t}$. Hence, the agent cannot accurately evaluate the posterior distribution of $\theta^{*}$ and sample from it as is conducted in standard contextual TS. Instead, our algorithm proposes to use a sampling distribution that ‘approximates’ the posterior.

Different from existing works that focus on frequentist regret analysis, we derive novel *information-theoretic* bounds on the *Bayesian cumulative regret* of our algorithm. For Gaussian bandits, our information-theoretic regret bounds scale as $\tilde{O}\left(m\sqrt{T}\right)$ (the notation $\tilde{O}\left(•\right)$ suppresses logarithmic terms in •), where m,T denote the dimension of the feature vector and time horizon respectively, under certain conditions on the variance of the prior on $\theta^{*}$. Furthermore, our Bayesian regret analysis shows that the *posterior mismatch*, resulting due to replacing the true posterior distribution with a sampling distribution, results in an approximation error that is captured via the Kullback–Leibler (KL) divergence between the distributions. To the best of our knowledge, quantifying the posterior mismatch via KL divergence has not been studied before and is of independent interest.

Finally, we also extend our algorithm to a setting where the agent observes the true context after the decision is made and reward is observed [12]. We call this setting CBs with delayed true contexts. Such scenarios arise in many applications where only a prediction of the context is available at the time of decision-making; however, the true context is available later. For instance, in farming-recommender systems where, at the time of making the decision regarding which crop to cultivate in a year, the true contextual information about the weather pattern is unavailable, while some ‘noisy’ weather predictions are available. In fact, the true weather pattern is observed only after the decision is made. We show that our TS algorithm for this setting with delayed true contexts results in reduced Bayesian regret. Table 1 compares our regret bound with that of the state-of-the-art algorithms in the noiseless and noisy CB settings.

## 2. Problem Setting

In this section, we present the stochastic linear CB problem studied in this paper. Let A denote the action set with *K* actions and C denote the (possibly infinite) set of *d*-dimensional context vectors. At iteration t∈N, the environment randomly draws a context vector $c_{t}\in C$ according to a *context distribution* P(c) defined over the space C of context vectors. The context distribution P(c) is known to the agent. The agent, however, does not observe the true context $c_{t}$ drawn by the environment. Instead, it observes a noisy version ${\hat{c}}_{t}$ of the true context, obtained as the output of a noisy, stochastic channel $P({\hat{c}}_{t}|c_{t},\gamma^{*})$ with the true context $c_{t}$ as the input. The noise channel $P({\hat{c}}_{t}|c_{t},\gamma^{*})$ is parameterized by the *noise channel parameter* $\gamma^{*}$ that is *unknown* to the agent.

Having observed the noisy context ${\hat{c}}_{t}$ at iteration *t*, the agent chooses an action $a_{t}\in A$ according to an *action policy* $\pi_{t}\left(\cdot |{\hat{c}}_{t}\right)$. The action policy may be stochastic describing a probability distribution over the set A of actions. Corresponding to the chosen action $a_{t}$, the agent receives a reward from the environment given by

(1) $$\begin{array}{c} r_{t}=f\left(\theta^{*},a_{t},c_{t}\right)+\xi_{t}, \end{array}$$

where $f\left(\theta^{*},a_{t},c_{t}\right)=\phi {\left(a_{t},c_{t}\right)}^{⊤}\theta^{*}$ is the linear *mean-reward function* and $\xi_{t}$ is a zero-mean reward noise variable. The mean reward function $f(\theta^{*},a_{t},c_{t})$ is defined via the *feature map* $\phi :A\times C\to R^{m}$, that maps the action and true context to an *m*-dimensional feature vector, and via the reward parameter $\theta^{*}\in R^{m}$ that is *unknown* to the agent.

We call the noisy CB problem described above *CBs with unobserved true context* (see Setting 1) since the agent does not observe the true context $c_{t}$ and the selection of action is based solely on the observed noisy context. Accordingly, at the end of iteration *t*, the agent has accrued the history $H_{t,r,a,\hat{c}}={\left\{r_{\tau },a_{\tau },{\hat{c}}_{\tau }\right\}}_{\tau =1}^{t}$ of observed reward-action-noisy context tuples. The action policy $\pi_{t+1}\left(\cdot |{\hat{c}}_{t+1}\right)$ at ${\left(t+1\right)}^{\mathrm{th}}$ iteration may depend on the history $H_{t,r,a,\hat{c}}$.
**Setting 1**: CBs with unobserved true contexts1:**for** t=1,…,T **do**2:    Environment samples $c_{t}∼P\left(c\right)$.3:    Agent observes noisy context ${\hat{c}}_{t}∼P\left({\hat{c}}_{t}|c_{t},\gamma^{*}\right)$.4:    Agent chooses an action $a_{t}∼\pi_{t}\left(\cdot |{\hat{c}}_{t}\right)$.5:    Agent receives reward $r_{t}$ according to (1).6:**end for**

We also consider a variant of the above problem where the agent has access to a *delayed* observation of the true context $c_{t}$ as studied in [12]. We call this setting *CBs with delayed true context*. In this setting, at iteration *t*, the agent first observes a noisy context ${\hat{c}}_{t}$, chooses action $a_{t}∼\pi_{t}\left(\cdot |{\hat{c}}_{t}\right)$, and receives reward $r_{t}$. Later, the true context $c_{t}$ is observed. It is important to note that the agent has no access to the true context at the time of decision-making. Thus, at the end of iteration *t*, the agent has collected the history $H_{t,r,a,c,\hat{c}}={\left\{r_{\tau },a_{\tau },c_{\tau },{\hat{c}}_{\tau }\right\}}_{\tau =1}^{t}$ of observed reward-action-context-noisy context tuples.

In both of the problem settings described above, the agent’s objective is to devise an action policy that minimizes the *Bayesian cumulative regret* with respect to a baseline action policy. We define Bayesian cumulative regret next.

### Bayesian Cumulative Regret

The cumulative regret of an action policy $\pi_{t}\left(\cdot |{\hat{c}}_{t}\right)$ quantifies how different the mean reward accumulated over *T* iterations is from that accrued by a baseline action policy $\pi_{t}^{*}\left(\cdot |{\hat{c}}_{t}\right)$. In this work, we consider as baseline the action policy of an *oracle* that has access to the channel noise parameter $\gamma^{*}$, reward parameter $\theta^{*}$, the context distribution P(c) and the noise channel likelihood $P(c_{t}|{\hat{c}}_{t},\gamma^{*}).$ Accordingly, at each iteration *t*, the oracle can infer the *exact predictive distribution* $P(c_{t}|{\hat{c}}_{t},\gamma^{*})$ of the true context from the observed noisy context ${\hat{c}}_{t}$ via Baye’s rule as

(2) $$\begin{array}{c} P\left(c_{t}|{\hat{c}}_{t},\gamma^{*}\right)=\frac{P(c_{t},{\hat{c}}_{t}|\gamma^{*})}{P({\hat{c}}_{t}|\gamma^{*})}. \end{array}$$

Here, $P\left(c_{t},{\hat{c}}_{t}|\gamma^{*}\right)=P\left(c_{t}\right)P\left({\hat{c}}_{t}|c_{t},\gamma^{*}\right)$ is the joint distribution of the true and noisy contexts given the noise channel parameter $\gamma^{*}$, and $P({\hat{c}}_{t}|\gamma^{*})$ is the distribution obtained by marginalizing $P(c_{t},{\hat{c}}_{t}|\gamma^{*})$ over the true contexts, i.e., 

(3) $$\begin{array}{c} P\left({\hat{c}}_{t}|\gamma^{*}\right)=E_{P(c_{t})}\left[P\left({\hat{c}}_{t}|c_{t},\gamma^{*}\right)\right], \end{array}$$

where $E_{•}\left[\cdot \right]$ denotes expectation with respect to ‘•’. The oracle action policy then adopts an action

(4) $$\begin{array}{cc} a_{t}^{*} & =\arg\max_{a\in A}E_{P(c_{t}|{\hat{c}}_{t},\gamma^{*})}\left[\phi {\left(a,c_{t}\right)}^{⊤}\theta^{*}\right]\\ \; & =\arg\max_{a\in A}\psi {\left(a,{\hat{c}}_{t}|\gamma^{*}\right)}^{⊤}\theta^{*}, \end{array}$$

at iteration *t*, where $\psi \left(a,\hat{c}|\gamma^{*}\right):=E_{P(c|\hat{c},\gamma^{*})}\left[\phi \left(a,c\right)\right]$. Note, that as in [14,18], we do not choose the stronger oracle action policy of $\arg\max_{a\in A}$$\phi {\left(a,c_{t}\right)}^{⊤}\theta^{*}$, that requires access to the true context $c_{t}$, as it is generally not achievable by an agent that observes only noisy context ${\hat{c}}_{t}$ and has no access to $\gamma^{*}$.

For fixed parameters $\theta^{*}$ and $\gamma^{*}$, we define the cumulative regret of the action policy $\pi_{t}\left(\cdot |{\hat{c}}_{t}\right)$ as

(5) $$\begin{array}{c} R^{T}\left(\pi |\theta^{*},\gamma^{*}\right)=\sum_{t=1}^{T}E\left[\phi {\left(a_{t}^{*},c_{t}\right)}^{⊤}\theta^{*}-\phi {\left(a_{t},c_{t}\right)}^{⊤}\theta^{*}|\theta^{*},\gamma^{*}\right], \end{array}$$

the expected difference in mean rewards of the oracle decision policy and the agent’s decision policy over *T* iterations. In (5), the expectation is taken with respect to the joint distribution $P\left(H_{t-1,r,\hat{c},c,a}|\theta^{*},\gamma^{*}\right)P\left({\hat{c}}_{t},c_{t},a_{t}|H_{t-1,r,\hat{c},c,a},\theta^{*},\gamma^{*}\right)$, where $P\left({\hat{c}}_{t},c_{t},a_{t}|H_{t-1,r,\hat{c},c,a},\theta^{*},\gamma^{*}\right)=P\left({\hat{c}}_{t},c_{t}|\gamma^{*}\right)\pi_{t}\left(a_{t}|{\hat{c}}_{t}\right)=P\left({\hat{c}}_{t}|\gamma^{*}\right)P\left(c_{t}|{\hat{c}}_{t},\gamma^{*}\right)\pi_{t}\left(a_{t}|{\hat{c}}_{t}\right)$. Using this, the cumulative regret (5) can be written as

(6) $$\begin{array}{cc} \; & R^{T}\left(\pi |\theta^{*},\gamma^{*}\right)\\ \; & =\sum_{t=1}^{T}E_{P(H_{t-1,r,\hat{c},c,a}|\theta^{*},\gamma^{*})}\left[E_{P\left({\hat{c}}_{t}|\gamma^{*}\right)\pi_{t}\left(a_{t}|{\hat{c}}_{t}\right)}E_{P(c_{t}|{\hat{c}}_{t},\gamma^{*})}\left[\phi {\left(a_{t}^{*},c_{t}\right)}^{⊤}\theta^{*}-\phi {\left(a_{t},c_{t}\right)}^{⊤}\theta^{*}\right]\right]\\ \; & =\sum_{t=1}^{T}E_{P(H_{t-1,r,\hat{c},c,a}|\theta^{*},\gamma^{*})}\left[E_{P\left({\hat{c}}_{t}|\gamma^{*}\right)\pi_{t}\left(a_{t}|{\hat{c}}_{t}\right)}\left[\psi {\left(a_{t}^{*},{\hat{c}}_{t}|\gamma^{*}\right)}^{⊤}\theta^{*}-\psi {\left(a_{t},{\hat{c}}_{t}|\gamma^{*}\right)}^{⊤}\theta^{*}\right]\right]\\ \; & :=\sum_{t=1}^{T}E\left[\psi {\left(a_{t}^{*},{\hat{c}}_{t}|\gamma^{*}\right)}^{⊤}\theta^{*}-\psi {\left(a_{t},{\hat{c}}_{t}|\gamma^{*}\right)}^{⊤}\theta^{*}|\theta^{*},\gamma^{*}\right]. \end{array}$$

Our focus in this work is on a *Bayesian framework* where we assume that the reward parameter $\theta^{*}\in \Theta$ and channel noise parameter $\gamma^{*}\in \Gamma$ are independently sampled by the environment from prior distributions $P(\theta^{*})$, defined on the set Θ of reward parameters, and $P(\gamma^{*})$, defined on the set Γ of channel noise parameters, respectively. The agent has knowledge of the prior distributions, the reward likelihood in (1) and the noise channel likelihood $P({\hat{c}}_{t}|c_{t},\gamma^{*})$, although it does not observe the sampled $\gamma^{*}$ and $\theta^{*}$. Using the above prior distributions, we define *Bayesian cumulative regret* of the action policy $\pi_{t}\left(\cdot |{\hat{c}}_{t}\right)$ as

(7) $$\begin{array}{c} R^{T}\left(\pi \right)=E\left[R^{T}\left(\pi |\theta^{*},\gamma^{*}\right)\right], \end{array}$$

where the expectation is taken with respect to the priors $P(\theta^{*})$ and $P(\gamma^{*})$.

In the next sections, we present our novel TS algorithms to minimize the Bayesian cumulative regret for the two problem settings considered in this paper.

## 3. Modified TS for CB with Unobserved True Contexts

In this section, we consider Setting 1 where the agent only observes the noisy context ${\hat{c}}_{t}$ at each iteration *t*. Our proposed modified TS Algorithm is given in Algorithm 1.
**Algorithm 1**: TS with unobserved true contexts ($\pi^{\mathrm{TS}}$)1:**for** t=1,…,T **do**2:    The environment selects a true context $c_{t}$.3:    Agent observes noisy context ${\hat{c}}_{t}$.4:    Agent evaluates the predictive posterior distribution $P(c_{t}|{\hat{c}}_{t},H_{t-1,\hat{c}})$ as in (8).5:    Agent samples $\theta_{t}∼\bar{P}\left(\theta^{*}|H_{t-1,r,a,\hat{c}}\right)$.6:    Agent chooses action $a_{t}$ as in (11).7:    Agent observes reward $r_{t}$ as in (1).8:**end for**

The proposed algorithm implements two steps in each iteration t∈N. In the first step, called the *de-noising* step, the agent uses the current observed noisy context ${\hat{c}}_{t}$ and the history $H_{t-1,\hat{c}}={\left\{{\hat{c}}_{\tau }\right\}}_{\tau =1}^{t-1}$ of past observed noisy contexts to obtain a *predictive posterior distribution* $P(c_{t}|{\hat{c}}_{t},H_{t-1,\hat{c}})$ of the true context $c_{t}$. This is a two-step process, where firstly the agent uses the history $H_{t-1,\hat{c}}$ of past observed noisy contexts to compute the posterior distribution of $\gamma^{*}$ as $P\left(\gamma^{*}|H_{t-1,\hat{c}}\right)\propto P\left(\gamma^{*}\right)\prod_{\tau =1}^{t-1}P\left({\hat{c}}_{\tau }|\gamma^{*}\right),$ where the conditional distribution $P({\hat{c}}_{t}|\gamma^{*})$ is evaluated as in (3). Note, that to evaluate the posterior, the agent uses its knowledge of the context distribution P(c), the prior $P(\gamma^{*})$ and the noise channel likelihood $P({\hat{c}}_{t}|c_{t},\gamma^{*})$. Using the derived posterior $P(\gamma^{*}|H_{t-1,\hat{c}})$, the predictive posterior distribution of the true context is then obtained as

(8) $$\begin{array}{c} P\left(c_{t}|{\hat{c}}_{t},H_{t-1,\hat{c}}\right)=E_{P(\gamma^{*}|H_{t-1,\hat{c}})}\left[P\left(c_{t}|{\hat{c}}_{t},\gamma^{*}\right)\right], \end{array}$$

where $P(c_{t}|{\hat{c}}_{t},\gamma^{*})$ is defined as in (2).

The second step of the algorithm implements a *modified* Thompson sampling. Note, that since the agent does not have access to the true contexts, it cannot evaluate the posterior distribution with known contexts,

(9) $$\begin{array}{c} P\left(\theta^{*}|H_{t-1,r,a,c}\right)\propto P\left(\theta^{*}\right)\prod_{\tau =1}^{t-1}P\left(r_{\tau }|a_{\tau },c_{\tau },\theta^{*}\right), \end{array}$$

as is conducted in standard contextual TS. Instead, the agent must evaluate the true *posterior distribution* under noisy contexts,

(10) $$\begin{array}{cc} P_{t}\left(\theta^{*}\right) & :=P(\theta^{*}|H_{t-1,r,a,\hat{c}})\\ & \propto P\left(\theta^{*}\right)E_{P(\gamma^{*})}\left[\prod_{\tau =1}^{t-1}E_{P(c_{\tau })}\left[P\left({\hat{c}}_{\tau }|c_{\tau },\gamma^{*}\right)P\left(r_{\tau }|a_{\tau },c_{\tau },\theta^{*}\right)\right]\right]. \end{array}$$

However, evaluating the marginal distribution $E_{P(\gamma^{*})}\left[\prod_{\tau =1}^{t-1}E_{P(c_{\tau })}\left[P\left({\hat{c}}_{\tau }|c_{\tau },\gamma^{*}\right)P\left(r_{\tau }|a_{\tau },c_{\tau },\theta^{*}\right)\right]\right]$ is challenging even for Gaussian bandits as the mean $\phi {\left(a_{\tau },c_{\tau }\right)}^{⊤}\theta^{*}$ of the reward distribution $P(r_{\tau }|a_{\tau },c_{\tau },\theta^{*})$ is, in general, a non-linear function of the true context $c_{\tau }$. As a result, the posterior $P_{t}\left(\theta^{*}\right)$ is analytically intractable.

Consequently, at each iteration *t*, the agent samples $\theta_{t}∼\bar{P}\left(\theta^{*}|H_{t-1,r,a,\hat{c}}\right)$ from a distribution $\bar{P}\left(\theta^{*}|H_{t-1,r,a,\hat{c}}\right)$ that ‘approximates’ the true posterior $P_{t}\left(\theta^{*}\right)$. The specific choice of this sampling distribution depends on the problem setting. Ideally, one must choose a distribution that is sufficiently ‘close’ to the true posterior. In the next sub-section, we will explain the choice for Gaussian bandits.

Using the sampled $\theta_{t}$ and the predictive posterior distribution $P(c_{t}|{\hat{c}}_{t},H_{t-1,\hat{c}})$ obtained from the denoising step, the agent then chooses action $a_{t}$ at iteration *t* as

(11) $$\begin{array}{cc} a_{t} & =\arg\max_{a\in A}\psi {\left(a,{\hat{c}}_{t}|H_{\hat{c}}\right)}^{⊤}\theta_{t},\mathrm{where} \end{array}$$

(12) $$\begin{array}{cc} \psi (a_{t},{\hat{c}}_{t}|H_{\hat{c}}) & :=E_{P(c_{t}|{\hat{c}}_{t},H_{t-1,\hat{c}})}\left[\phi \left(a_{t},c_{t}\right)\right] \end{array}$$

is the expected feature map with respect to $P(c_{t}|{\hat{c}}_{t},H_{t-1,\hat{c}})$.

### 3.1. Linear-Gaussian Stochastic CBs

We now instantiate Algorithm 1 for Gaussian CBs. Specifically, we consider Gaussian bandits with the reward noise $\xi_{t}$ in (1) as Gaussian $N(0,\sigma^{2})$ with mean 0 and variance $\sigma^{2}>0$. We also assume a Gaussian prior $P\left(\theta^{*}\right)=N\left(0,\lambda I\right)$ on the reward parameter $\theta^{*}$ with mean zero and an m×m diagonal, covariance matrix with entries λ>0. Here, I denotes the identity matrix. The assumption of diagonal prior covariance is in line with Lemma 3 in [19].

We consider a multivariate Gaussian context distribution $P\left(c\right)=N\left(\mu_{c},\Sigma_{c}\right)$ with mean $\mu_{c}\in R^{d}$ and covariance matrix $\Sigma_{c}\in R^{d\times d}$. The context noise channel $P(\hat{c}|c,\gamma^{*})$ is also similarly Gaussian with a mean $\left(\gamma^{*}+c\right)$ and covariance matrix $\Sigma_{n}\in R^{d\times d}$. We assume the prior on noise channel parameter $\gamma^{*}$ to be Gaussian $P\left(\gamma^{*}\right)=N\left(0,\Sigma_{\gamma }\right)$ with *d*-dimensional zero mean vector 0 and covariance matrix $\Sigma_{\gamma }\in R^{d\times d}.$ We assume that $\Sigma_{c},\Sigma_{\gamma }$ and $\Sigma_{n}$ are all positive definite matrices known to the agent. The assumption of positive definite covariance matrices is to facilitate the Bayesian analysis adopted in this work. Similar assumptions were also required in the related work of [14].

For this setting, we can analytically evaluate the predictive posterior distribution $P\left(c_{t}|{\hat{c}}_{t},H_{t-1,\hat{c}}\right)=N\left(c_{t}|V_{t},R_{t}^{-1}\right)$ as a multi-variate Gaussian with inverse covariance matrix,

(13) $$\begin{array}{c} R_{t}=M-\Sigma_{n}^{-1}{\left(H_{t}^{-1}\right)}^{⊤}\Sigma_{n}^{-1}, \end{array}$$

where $H_{t}=\left(t-1\right)\Sigma_{n}^{-1}-\left(t-2\right)\Sigma_{n}^{-1}M^{-1}\Sigma_{n}^{-1}+\Sigma_{\gamma }^{-1}$ and $M=\Sigma_{c}^{-1}+\Sigma_{n}^{-1}$, and with the mean vector

(14) $$\begin{array}{c} V_{t}={\left(R_{t}^{-1}\right)}^{⊤}\left(\Sigma_{c}^{-1}\mu_{c}+\Sigma_{n}^{-1}{\hat{c}}_{t}-\Sigma_{n}^{-1}{\left(H_{t}^{-1}\right)}^{⊤}L_{t}^{⊤}\right), \end{array}$$

where

$$\begin{array}{cc} L_{t}^{⊤} & =\Sigma_{n}^{-1}M^{-1}\left(\Sigma_{c}^{-1}\mu_{c}+\Sigma_{n}^{-1}{\hat{c}}_{t}\right)+\left(\Sigma_{n}^{-1}-\Sigma_{n}^{-1}M^{-1}\Sigma_{n}^{-1}\right)\sum_{\tau =1}^{t-1}{\hat{c}}_{\tau }\\ & -\left(t-1\right)\Sigma_{n}^{-1}M^{-1}\Sigma_{c}^{-1}\mu_{c}. \end{array}$$

Derivations are presented in Appendix C.1.2.

For the *modified*-TS step, we sample $\theta_{t}$ from the approximate posterior distribution

(15) $$\begin{array}{c} {\bar{P}}_{t}\left(\theta^{*}\right):=\bar{P}\left(\theta^{*}|H_{t-1,r,a,\hat{c}}\right)\propto P\left(\theta^{*}\right)\prod_{\tau =1}^{t-1}\bar{P}\left(r_{\tau }|a_{\tau },{\hat{c}}_{\tau },H_{\tau -1,\hat{c}},\theta^{*}\right), \end{array}$$

where

(16) $$\begin{array}{c} \bar{P}\left(r_{\tau }|a_{\tau },{\hat{c}}_{\tau },H_{\tau -1,\hat{c}},\theta^{*}\right)=N\left(\psi {\left(a_{\tau },{\hat{c}}_{\tau }|H_{\hat{c}}\right)}^{⊤}\theta^{*},\sigma^{2}\right) \end{array}$$

and $\psi (a_{t},{\hat{c}}_{t}|H_{\hat{c}})$ is the expected feature map defined in (12). This yields the approximate posterior to be a Gaussian distribution ${\bar{P}}_{t}\left(\theta^{*}\right)=N\left(\mu_{t-1},\Sigma_{t-1}^{-1}\right)$ whose inverse covariance matrix and mean, respectively, evaluate as

(17) $$\begin{array}{cc} \Sigma_{t-1} & =\frac{I}{\lambda }+\frac{1}{\sigma^{2}}\sum_{\tau =1}^{t-1}\psi \left(a_{\tau },{\hat{c}}_{\tau }|H_{\hat{c}}\right)\psi {\left(a_{\tau },{\hat{c}}_{\tau }|H_{\hat{c}}\right)}^{⊤} \end{array}$$

(18) $$\begin{array}{cc} \mu_{t-1} & =\frac{\Sigma_{t-1}^{-1}}{\sigma^{2}}\left(\sum_{\tau =1}^{t-1}r_{\tau }\psi \left(a_{\tau },{\hat{c}}_{\tau }|H_{\hat{c}}\right)\right). \end{array}$$

The sampling distribution ${\bar{P}}_{t}\left(\theta^{*}\right)$ considered above is different from the true posterior distribution (10), which is analytically intractable. However, it bears resemblance to the posterior (9) when the true contexts are known, with the reward distribution $P(r_{\tau }|a_{\tau },c_{\tau },\theta^{*})$ replaced by $\bar{P}\left(r_{\tau }|a_{\tau },{\hat{c}}_{\tau },H_{\tau -1,\hat{c}},\theta^{*}\right)$. In Section 3.2.2, we show that the above choice of sampling distribution is indeed ‘close’ to the true posterior.

### 3.2. Bayesian Regret Analysis

In this section, we derive information-theoretic upper bounds on the Bayesian regret (7) of the modified TS algorithm for Gaussian CBs. To this end, we first outline the key information-theoretic tools required to derive our bound.

#### 3.2.1. Preliminaries

To start, let P(x) and Q(x) denote two probability distributions defined over the space X of random variables *x*. Then, the Kullback–Leibler (KL)-divergence between the distributions P(x) and Q(x) is defined as

(19) $$\begin{array}{c} D_{\mathrm{KL}}\left(P\left(x\right)||Q\left(x\right)\right)=E_{P(x)}\left[\log\frac{P(x)}{Q(x)}\right], \end{array}$$

if P(x) is absolutely continuous with respect to Q(x), and takes value *∞* otherwise. If *x* and *y* denote two random variables described by the joint probability distribution P(x,y), the mutual information I(x;y) between *x* and *y* is defined as $I\left(x;y\right)=D_{\mathrm{KL}}\left(P\left(x,y\right)∥P\left(x\right)P\left(y\right)\right)$, where P(x) (and P(y)) is the marginal distribution of *x* (and *y*). More generally, for three random variables *x*, *y* and *z* with joint distribution P(x,y,z), the conditional mutual information I(x;y|z) between *x* and *y* given *z* evaluates as

$$I\left(x;y|z\right)=E_{P(z)}\left[D_{KL}\left(P\left(x,y|z\right)∥P\left(x|z\right)P\left(y|z\right)\right)\right]$$

where P(x|z) and P(y|z) are conditional distributions. We will also use the following variational representation of the KL-divergence, also termed the *Donskar–Varadhan* (*DV*) inequality,

(20) $$\begin{array}{c} D_{\mathrm{KL}}\left(P\left(x\right)∥Q\left(x\right)\right)\geq E_{P(x)}\left[f\left(x\right)\right]-\log E_{Q(x)}\left[\exp\left(f\left(x\right)\right)\right], \end{array}$$

which holds for any measurable function f:X→R satifying the inequality $E_{Q(x)}\left[\exp\left(f\left(x\right)\right)\right]$<∞.

#### 3.2.2. Information-Theoretic Bayesian Regret Bounds

In this section, we present information-theoretic upper bounds on the Bayesian regret of the modified TS algorithm. To this end, we first state our main assumption.

**Assumption** **1.** *The feature map $\phi \left(\cdot ,\cdot \right)\in R^{m}$ has bounded norm, i.e., ${∥\phi \left(\cdot ,\cdot \right)∥}_{2}\leq 1$.*

The following theorem gives our main result.

**Theorem** **1.** *Assume that the covariance matrices satisfy $\Sigma_{n}\Sigma_{c}^{-1}≻0$ and $\Sigma_{n}\Sigma_{\gamma }^{-1}\Sigma_{n}M≻0$ where $M=\Sigma_{n}^{-1}+\Sigma_{c}^{-1}$. Under Assumption 1, if $\frac{\lambda }{\sigma^{2}}\leq \frac{1}{T}\leq 1$, the following upper bound on the Bayesian regret of the modified TS algorithm holds,*

$$\begin{array}{cc} \; & R^{T}\left(\pi^{\mathrm{TS}}\right)\leq U\left(m,\frac{\sigma^{2}}{T}\right)+\sqrt{2Tm\sigma^{2}}+\sqrt{2T\sigma^{2}\left(\log\left(K\right)+m\right)}\\ & +\frac{4}{T}\sqrt{\frac{m\sigma^{2}}{2T\pi }}+2\sqrt{4\sigma^{2}m\log\left(2mT\right)\left(\mathrm{Tr}\left({\left(\Sigma_{n}\Sigma_{\gamma }^{-1}\Sigma_{n}M\right)}^{-1}\right)+\log\left(T\right)\mathrm{Tr}\left(\Sigma_{c}\Sigma_{n}^{-1}\right)\right)}, \end{array}$$

*where*

(21) $$\begin{array}{cc} U(m,\lambda ) & =\sqrt{2Tm\sigma^{2}\min\left\{m,2\log\left(1+K\right)\right\}\log\left(1+\frac{T\lambda }{m\sigma^{2}}\right)}. \end{array}$$

The theorem above shows that the proposed TS algorithm achieves $\tilde{O}\left(m\sqrt{T}\right)$ regret when the prior $P(\theta^{*})$ is highly informative with variance parameter satisfying the constraint $\lambda \leq \sigma^{2}/T$.

**Remark** **1.** *The assumption on covariance matrices in Theorem 1 directly holds for diagonal covariance matrices with positive eigen values.*

To prove the regret bound of Theorem 1, we start by defining

(22) $$\begin{array}{c} {\hat{a}}_{t}=\arg\max_{a\in A}\psi {\left(a,{\hat{c}}_{t}|H_{\hat{c}}\right)}^{⊤}\theta^{*} \end{array}$$

as the action that maximizes the mean reward $\psi {\left(a,{\hat{c}}_{t}|H_{\hat{c}}\right)}^{⊤}\theta^{*}$ corresponding to reward parameter $\theta^{*}$. Using the above, the Bayesian cumulative regret (7) for the proposed TS algorithm $\pi^{\mathrm{TS}}$ can be decomposed as

(23) $$\begin{array}{cc} \; & R^{T}\left(\pi^{\mathrm{TS}}\right)=R_{\mathrm{CB}}^{T}+R_{\mathrm{EE}1}^{T}+R_{\mathrm{EE}2}^{T},\mathrm{where}\\ \; & R_{\mathrm{CB}}^{T}=\sum_{t=1}^{T}E\left[\psi {\left({\hat{a}}_{t},{\hat{c}}_{t}|H_{\hat{c}}\right)}^{⊤}\theta^{*}-\psi {\left(a_{t},{\hat{c}}_{t}|H_{\hat{c}}\right)}^{⊤}\theta^{*}\right],\\ & R_{\mathrm{EE}1}^{T}=\sum_{t=1}^{T}E\left[\psi {\left(a_{t}^{*},{\hat{c}}_{t}|\gamma^{*}\right)}^{⊤}\theta^{*}-\psi {\left({\hat{a}}_{t},{\hat{c}}_{t}|H_{\hat{c}}\right)}^{⊤}\theta^{*}\right],\\ & R_{\mathrm{EE}2}^{T}=\sum_{t=1}^{T}E\left[\psi {\left(a_{t},{\hat{c}}_{t}|H_{\hat{c}}\right)}^{⊤}\theta^{*}-\psi {\left(a_{t},{\hat{c}}_{t}|\gamma^{*}\right)}^{⊤}\theta^{*}\right]. \end{array}$$

In (23), the first term $R_{\mathrm{CB}}^{T}$ quantifies the Bayesian regret of our action policy (11) with respect to the action policy (22) for a CB with mean reward function $\psi {\left(a,{\hat{c}}_{t}|H_{\hat{c}}\right)}^{⊤}\theta^{*}$. The second term $R_{\mathrm{EE}1}^{T}$ accounts for the average difference in the cumulative mean rewards of the oracle optimal action policy (4), evaluated using the exact predictive distribution $P(c_{t}|{\hat{c}}_{t},\gamma^{*})$, and our action policy (11), that uses the inferred predictive posterior distribution $P(c_{t}|{\hat{c}}_{t},H_{t-1,\hat{c}})$. In this sense, $R_{\mathrm{EE}1}^{T}$ captures the error in approximating the exact predictive distribution $P(c_{t}|{\hat{c}}_{t},\gamma^{*})$ via the inferred predictive distribution $P(c_{t}|{\hat{c}}_{t},H_{\hat{c}})$. The third term $R_{\mathrm{EE}2}^{T}$ similarly accounts for the average approximation error.

To derive an upper bound on the Bayesian regret $R^{T}\left(\pi^{\mathrm{TS}}\right)$, we separately upper bound each of the three terms in (23) as derived in the following lemmas. The lemma below presents an upper bound on $R_{\mathrm{CB}}^{T}$.

**Lemma** **1.** *Under Assumption 1, the following upper bound holds if $\frac{\lambda }{\sigma^{2}}\leq \frac{1}{T}\leq 1$,*

(24) $$\begin{array}{cc} R_{\mathrm{CB}}^{T} & \leq U\left(m,\frac{\sigma^{2}}{T}\right)+\sqrt{2\sigma^{2}\sum_{t=1}^{T}D_{t}}+\sqrt{2\sigma^{2}\left(T\log\left(K\right)+\sum_{t=1}^{T}D_{t}\right)}, \end{array}$$

(25) $$\begin{array}{cc} \; & \leq U\left(m,\frac{\sigma^{2}}{T}\right)+\sqrt{2Tm\sigma^{2}}+\sqrt{2T\sigma^{2}\left(\log\left(K\right)+m\right)}, \end{array}$$

where $D_{t}=E\left[D_{\mathrm{KL}}\left(P_{t}\left(\theta^{*}\right)∥{\bar{P}}_{t}\left(\theta^{*}\right)\right)\right]$ and U(m,λ) is as defined in (21).

To derive the upper bound in (24), we leverage results from [19] that study information-theoretic Bayesian regret of standard contextual TS algorithms via lifted information-ratio. However, the results do not directly apply to our algorithm due to the *posterior mismatch* between the sampling distribution ${\bar{P}}_{t}\left(\theta^{*}\right)$ and the true posterior distribution $P_{t}\left(\theta^{*}\right)$. Consequently, our upper bound (24) consists of three terms: the first term, defined as in (21), corresponds to the upper bound on the Bayesian regret of contextual TS that assumes ${\bar{P}}_{t}\left(\theta^{*}\right)$ as the true posterior. This can be obtained by applying the lifted information ratio-based analysis of Cor. 2 in [19]. The second and third terms account for the posterior mismatch via the expected KL-divergence $E[D_{\mathrm{KL}}\left(P_{t}\left(\theta^{*}\right)∥{\bar{P}}_{t}\left(\theta^{*}\right)\right)]$ between the true posterior $P_{t}\left(\theta^{*}\right)$ and the sampling distribution ${\bar{P}}_{t}\left(\theta^{*}\right)$. In particular, this expected KL divergence can be upper bounded by $2\left(t-1\right)\lambda m/\sigma^{2}$ (See Appendix C.1.3 for proof) under the prior $P\left(\theta^{*}\right)=N\left(0,\lambda I\right)$. Importantly, our result holds when this prior distribution is sufficiently concentrated with its variance satisfying the inequality $\lambda \leq \sigma^{2}/T$. This ensures that the contribution of posterior mismatch to the Bayes regret scales is $O(\sqrt{mT})$.

The following lemma gives an upper bound on the sum $R_{\mathrm{EE}1}^{T}+R_{\mathrm{EE}2}^{T}$.

**Lemma** **2.** *Under Assumption 1, the following upper bound holds for δ∈(0,1),*

(26) $$\begin{array}{ll} R_{\mathrm{EE}1}^{T}+R_{\mathrm{EE}2}^{T} & \leq 2R_{\mathrm{EE}1}^{T}\\ & \leq 4\delta^{2}T\sqrt{\frac{m\lambda }{2\pi }}+2\sqrt{4\lambda Tm\log\left(\frac{2m}{\delta }\right)\sum_{t=1}^{T}I\left(\gamma^{*};c_{t}|{\hat{c}}_{t},H_{t-1,\hat{c}}\right)}. \end{array}$$

*In addition, if the covariance matrices satisfy that $\Sigma_{n}\Sigma_{c}^{-1}≻0$ and $\Sigma_{n}\Sigma_{\gamma }^{-1}\Sigma_{n}M≻0$ where $M=\Sigma_{n}^{-1}+\Sigma_{c}^{-1}$, then* (26) *can be further upper bounded as*

(27) $$\begin{array}{cc} R_{\mathrm{EE}1}^{T}+R_{\mathrm{EE}2}^{T} & \\ & \leq 4\delta^{2}T\sqrt{\frac{m\lambda }{2\pi }}+2\sqrt{4\lambda Tm\log\left(\frac{2m}{\delta }\right)\left(\mathrm{Tr}\left({\left(\Sigma_{n}\Sigma_{\gamma }^{-1}\Sigma_{n}M\right)}^{-1}\right)+\log\left(T\right)\mathrm{Tr}\left(\Sigma_{c}\Sigma_{n}^{-1}\right)\right)}. \end{array}$$

Lemma 2 shows that the error in approximating $P(c_{t}|{\hat{c}}_{t},\gamma^{*})$ with $P(c_{t}|{\hat{c}}_{t},H_{t-1,\hat{c}})$, on average, can be quantified via the conditional mutual information $I(\gamma^{*};c_{t}|{\hat{c}}_{t},H_{t-1,\hat{c}})$ between $\gamma^{*}$ and true context $c_{t}$ given knowledge of observed noisy contexts up to and including iteration *t*.

Finally, combining Lemmas 1 and  2 with the choice of δ=1/T and $\lambda \leq \sigma^{2}/T$ gives us the regret bound in Theorem 1.

### 3.3. Beyond Gaussian Bandits

In the previous sections, we studied Gaussian bandits and analyzed the Bayesian regret. We will now discuss the potential extension of results beyond Gaussian bandits. As in [14], we will focus on Gaussian context distribution and context noise distribution, which helps to derive the upper bound on the estimation errors in Lemma 2.

To extend the Bayesian regret analysis to non-Gaussian bandits, Lemma 1 requires bandit-specific modifications. Specifically, the derivation of the term U(m,λ), that captures the standard Bayesian regret of contextual TS with ${\bar{P}}_{t}\left(\theta^{*}\right)$ as the true posterior, and that of the posterior mismatch term via the expected KL divergence critically depends on the type of bandit and the choice of the sampling posterior. The Bayesian regret bound U(m,λ) is derived using the lifted information ratio-based approach of [19]. This can indeed be extended to non-Gaussian bandits like logistic bandits (see [19]) to obtain a modified U(m,λ) term.

However, the analysis of posterior mismatch term for non-Gaussian bandits is non-trivial and depends on the specific bandit assumed. Firstly, to characterize the posterior mismatch via the expected KL divergence, our analysis requires the chosen sampling distribution ${\bar{P}}_{t}\left(\theta^{*}\right)$ to be sub-Gaussian. To choose the sampling distribution, one can follow the framework adopted in (15) and (16) and use an ‘appropriate’ reward distribution $\bar{P}\left(r_{\tau }|a_{\tau },{\hat{c}}_{\tau },H_{\tau -1,\hat{c}},\theta^{*}\right)$ such that (a) the KL divergence $D_{\mathrm{KL}}\left(P\left(r_{\tau }|a_{\tau },c_{\tau },\theta^{*}\right)∥\bar{P}\left(r_{\tau }|a_{\tau },{\hat{c}}_{\tau },H_{\tau -1,\hat{c}},\theta^{*}\right)\right)$ between the true reward distribution and the chosen reward distribution is small to minimize posterior mismatch, and  (b) the resulting sampling distribution is easy to sample from and has sub-Gaussian tails. Thus, analyzing the posterior mismatch for non-Gaussian bandits requires a case-by-case treatment. For Gaussian bandits, we control the above KL divergence by choosing a Gaussian distribution $\bar{P}\left(r_{\tau }|a_{\tau },{\hat{c}}_{\tau },H_{\tau -1,\hat{c}},\theta^{*}\right)$ with mean $\psi (a_{\tau },{\hat{c}}_{\tau }|H_{\hat{c}})$ as in (16). Finally, in Section 5, we extend Algorithm 1 to logistic bandits with the choice of sampling distribution motivated by (15) and (16) and use Langevin Monte Carlo to sample from this distribution.

## 4. TS for CB with Delayed True Contexts

In this section, we consider the CBs with delayed true context setting where the agent observes the true context $c_{t}$ after it observes the reward $r_{t}$ corresponding to the chosen action $a_{t}$. Note, that at the time of choosing action $a_{t}$, the agent has access only to noisy contexts. We specialize our TS algorithm to this setting, and call it Algorithm 2 (or $\pi_{\mathrm{delay}}^{\mathrm{TS}}$).
**Algorithm 2**: TS for Delayed True contexts ($\pi_{\mathrm{delay}}^{\mathrm{TS}}$)1:**for** t=1,…,T **do**2:    The environment selects a true context $c_{t}$.3:    Agent observes noisy context ${\hat{c}}_{t}$.4:    Agent evaluates the predictive posterior distribution $P(c_{t}|{\hat{c}}_{t},H_{t-1,c,\hat{c}})$ as in (28).5:    Agent samples $\theta_{t}∼P\left(\theta^{*}|H_{t-1,r,a,c}\right)$.6:    Agent chooses action $a_{t}$ as in (33).7:    Agent observes reward $r_{t}$ (as in (1)) and the true context $c_{t}$.8:**end for**

Algorithm 2 follows similar steps as in Algorithm 1. However, different from Algorithm 1, at the *t*th iteration, the agent knows the history $H_{t-1,c,\hat{c}}$ of true contexts in addition to that of noisy contexts. Consequently, in the *de-noising* step, the agent evaluates the predictive posterior distribution as

(28) $$\begin{array}{c} P\left(c_{t}|{\hat{c}}_{t},H_{t-1,c,\hat{c}}\right)=E_{P(\gamma^{*}|H_{t-1,c,\hat{c}})}\left[P\left(c_{t}|{\hat{c}}_{t},\gamma^{*}\right)\right], \end{array}$$

where $P(c_{t}|{\hat{c}}_{t},\gamma^{*})$ is as defined in (2) and posterior distribution $P(\gamma^{*}|H_{t-1,c,\hat{c}})$ is obtained via Baye’s rule as $P\left(\gamma^{*}|H_{t-1,c,\hat{c}}\right)\propto P\left(\gamma^{*}\right)\prod_{\tau =1}^{t-1}P\left(c_{\tau },{\hat{c}}_{\tau }|\gamma^{*}\right)$ using the history of true and noisy contexts.

For the Gaussian context, noise as considered in Section 3.1, the predictive posterior distribution $P\left(c_{t}|{\hat{c}}_{t},H_{t-1,c,\hat{c}}\right)=N\left({\tilde{V}}_{t},{\tilde{R}}_{t}^{-1}\right)$ is multivariate Gaussian with the inverse covariance matrix,

(29) $$\begin{array}{cc} \tilde{R_{t}} & =M-\Sigma_{n}^{-1}{\tilde{H}}_{t}^{-1}\Sigma_{n}^{-1}, \end{array}$$

and the mean vector

(30) $$\begin{array}{cc} \tilde{V_{t}} & ={\tilde{R}}_{t}^{-1}\left(\Sigma_{c}^{-1}\mu_{c}+\Sigma_{n}^{-1}{\hat{c}}_{t}+\Sigma_{n}^{-1}{\tilde{H}}_{t}^{-1}\Sigma_{n}^{-1}\sum_{\tau =1}^{t-1}\left({\hat{c}}_{\tau }-c_{\tau }\right)-\Sigma_{n}^{-1}{\tilde{H}}_{t}^{-1}\Sigma_{n}^{-1}M^{-1}\left(\Sigma_{c}^{-1}\mu_{c}-\Sigma_{n}^{-1}{\hat{c}}_{t}\right)\right), \end{array}$$

where $M=\Sigma_{c}^{-1}+\Sigma_{n}^{-1}$ and ${\tilde{H}}_{t}=\Sigma_{n}^{-1}M^{-1}\Sigma_{n}^{-1}+\left(t-1\right)\Sigma_{n}^{-1}+\Sigma_{\gamma }^{-1}$. Derivation can be found in Appendix B.2.4.

Following the denoising step, the next step in Algorithm 2 is a conventional Thompson sampling step, thanks to access to delayed true contexts. Consequently, the agent can evaluate the posterior distribution $P(\theta^{*}|H_{t-1,r,a,c})$ with known contexts as in (9) and use it to sample $\theta_{t}∼P\left(\theta^{*}|H_{t-1,r,a,c}\right)$. For  Gaussian bandit with Gaussian prior on $\theta^{*}$, the posterior distribution $P\left(\theta^{*}|H_{t-1,r,a,c}\right)=N\left({\tilde{\mu }}_{t-1},{\tilde{\Sigma }}_{t-1}^{-1}\right)$ is a multivariate Gaussian distribution whose inverse covariance matrix and mean, respectively, evaluate as

(31) $$\begin{array}{cc} {\tilde{\Sigma }}_{t-1} & =\frac{1}{\lambda }I+\frac{1}{\sigma^{2}}\sum_{\tau =1}^{t-1}\phi \left(a_{\tau },c_{\tau }\right)\phi {\left(a_{\tau },c_{\tau }\right)}^{⊤} \end{array}$$

(32) $$\begin{array}{cc} {\tilde{\mu }}_{t-1} & =\frac{{\tilde{\Sigma }}_{t-1}^{-1}}{\sigma^{2}}\left(\sum_{\tau =1}^{t-1}r_{\tau }\phi \left(a_{\tau },c_{\tau }\right)\right). \end{array}$$

Using the sampled $\theta_{t}$ and the obtained predictive posterior distribution $P(c_{t}|{\hat{c}}_{t},H_{t-1,c,\hat{c}})$, the agent then chooses action $a_{t}$ as

(33) $$\begin{array}{cc} a_{t} & =\arg\max_{a\in A}\psi {\left(a,{\hat{c}}_{t}|H_{c,\hat{c}}\right)}^{⊤}\theta_{t}, \end{array}$$

where we use the expected feature map $\psi \left(a_{t},{\hat{c}}_{t}|H_{c,\hat{c}}\right):=E_{P(c_{t}|{\hat{c}}_{t},H_{t-1,c,\hat{c}})}\left[\phi \left(a_{t},c_{t}\right)\right]$.

### Information-Theoretic Bayesian Regret Bounds

In this section, we derive an information-theoretic upper bound on the Bayesian regret (7) of Algorithm 2 for Gaussian CBs. The following theorem presents our main result.

**Theorem** **2.** *Under Assumption 1 and assuming that covariance matrices satisfy $\Sigma_{\gamma }\Sigma_{n}^{-1}≻0$, the following inequality holds for δ∈(0,1) when $\lambda \leq \sigma^{2}$,*

$$\begin{array}{cc} \; & R^{T}\left(\pi_{\mathrm{delay}}^{\mathrm{TS}}\right)\leq U\left(m,\lambda \right)+4T\delta^{2}\sqrt{\frac{2m\lambda }{\pi }}+2\sqrt{2\lambda mTd\log\left(1+T\mathrm{Tr}(\Sigma_{\gamma }\Sigma_{n}^{-1})/d\right)\log\left(\frac{2m}{\delta }\right)}, \end{array}$$

*where U(m,λ) is as defined in* (21).

Theorem 2 shows that Algorithm 2 achieves $\tilde{O}\left(m\sqrt{T}\right)$ regret with the choice of δ=1/T if d=O(m). Furthermore, due to the absence of posterior mistmatch, the upper bound above is tighter than that of Theorem 1.

We now outline the main lemmas required to prove Theorem 2. To this end, we re-use the notation

(34) $$\begin{array}{c} {\hat{a}}_{t}=\arg\max_{a\in A}\psi {\left(a,{\hat{c}}_{t}|H_{c,\hat{c}}\right)}^{⊤}\theta^{*} \end{array}$$

to define the optimal action maximizing the mean reward $\psi {\left(a,{\hat{c}}_{t}|H_{c,\hat{c}}\right)}^{⊤}\theta^{*}$.

To derive the regret upper bound in Theorem 2, we first decompose the Bayesian cumulative regret (7) of Algorithm 2 ($\pi_{\mathrm{delay}}^{\mathrm{TS}}$), similar to (23), into the following three terms,

(35) $$\begin{array}{cc} \; & R^{T}\left(\pi_{\mathrm{delay}}^{\mathrm{TS}}\right)=R_{d,\mathrm{CB}}^{T}+R_{d,\mathrm{EE}1}^{T}+R_{d,\mathrm{EE}2}^{T}\mathrm{where},\\ & R_{d,\mathrm{CB}}^{T}=\sum_{t=1}^{T}E\left[\psi {\left({\hat{a}}_{t},{\hat{c}}_{t}|H_{c,\hat{c}}\right)}^{⊤}\theta^{*}-\psi {\left(a_{t},{\hat{c}}_{t}|H_{c,\hat{c}}\right)}^{⊤}\theta^{*}\right],\\ & R_{d,\mathrm{EE}1}^{T}=\sum_{t=1}^{T}E\left[\psi {\left(a_{t}^{*},{\hat{c}}_{t}|\gamma^{*}\right)}^{⊤}\theta^{*}-\psi {\left({\hat{a}}_{t},{\hat{c}}_{t}|H_{c,\hat{c}}\right)}^{⊤}\theta^{*}\right],\\ & R_{d,\mathrm{EE}2}^{T}=\sum_{t=1}^{T}E\left[\psi {\left(a_{t},{\hat{c}}_{t}|H_{c,\hat{c}}\right)}^{⊤}\theta^{*}-\psi {\left(a_{t},{\hat{c}}_{t}|\gamma^{*}\right)}^{⊤}\theta^{*}\right]. \end{array}$$

An upper bound on $R^{T}\left(\pi_{\mathrm{delay}}^{\mathrm{TS}}\right)$ can be then obtained by separately bounding each of the three terms in (35).

In (35), the first term $R_{d,\mathrm{CB}}^{T}$ corresponds to the Bayesian cumulative regret of a standard contextual TS algorithm that uses $\psi {\left(a,{\hat{c}}_{t}|H_{c,\hat{c}}\right)}^{⊤}\theta^{*}$ for a∈A as the mean reward function. Note, that due to availability of delayed true contexts, there is no posterior mismatch in Algorithm 2. Hence, we apply Cor. 3 in [19] to yield the following upper bound on $R_{d,\mathrm{CB}}^{T}$.

**Lemma** **3.** *Under Assumption 1, the following upper bound on $R_{d,\mathrm{CB}}^{T}$ holds for $\frac{\lambda }{\sigma^{2}}\leq 1$,*

(36) $$\begin{array}{cc} R_{d,\mathrm{CB}}^{T} & \leq U(m,\lambda ), \end{array}$$

*where U(m,λ) is defined as in* (21).

Lemma 3 gives a tighter bound in comparison to Lemma 1 where the posterior mismatch results in additional error terms in the regret bound.

We now upper bound the second term $R_{d,\mathrm{EE}1}^{T}$ of (35), which similar to the term $R_{\mathrm{EE}1}^{T}$ in (23), captures the error in approximating the exact predictive distribution $P(c_{t}|{\hat{c}}_{t},\gamma^{*})$ via the inferred predictive distribution $P(c_{t}|{\hat{c}}_{t},H_{c,\hat{c}})$. The following lemma shows that this approximation error over *T* iterations can be quantified, on average, via the mutual information $I(\gamma^{*};H_{T,c,\hat{c}})$ between $\gamma^{*}$ and the *T*-length history of observed true and noisy contexts. This bound also holds for the third term $R_{d,\mathrm{EE}2}^{T}$ of (35) which similarly accounts for the average approximation error.

**Lemma** **4.** *Under Assumption 1, for any δ∈(0,1), we have the following upper bound,*

$$\begin{array}{cc} \; & R_{d,\mathrm{EE}1}^{T}+R_{d,\mathrm{EE}2}^{T}\leq 2R_{d,\mathrm{EE}1}^{T}\leq 4\sqrt{m\lambda T\log\left(\frac{2m}{\delta }\right)I\left(\gamma^{*};H_{T,c,\hat{c}}\right)}+4T\delta^{2}\sqrt{\frac{2m\lambda }{\pi }}. \end{array}$$

*Furthermore, if the covariance matrices satisfy that $\Sigma_{\gamma }\Sigma_{n}^{-1}≻0$, we obtain that*

$$\begin{array}{c} I\left(\gamma^{*};H_{T,c,\hat{c}}\right)\leq \frac{d}{2}\log\left(1+T\mathrm{Tr}(\Sigma_{\gamma }\Sigma_{n}^{-1})/d\right)). \end{array}$$

Combining Lemmas 3 and  4 then gives us the upper bound on $R^{T}\left(\pi_{\mathrm{delay}}^{\mathrm{TS}}\right)$ in Theorem 1.

## 5. Experiments and Final Remarks

In this section, we experimentally validate the performance of the proposed algorithms on synthetic and real-world datasets. Details of implementation can be found in Appendix D.

**Figure 1:**
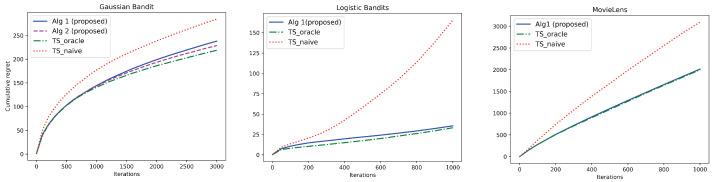
Comparison of Bayesian regret of proposed algorithms with baselines as a function of number of iterations. (**Left**): Gaussian bandits with K=40, $\sigma_{n}^{2}=\sigma_{\gamma }^{2}=1.1$; (**Center**) Logistic bandits with K=40, $\sigma_{n}^{2}=2$, $\sigma_{\gamma }^{2}=2.5$; (**Right**) MovieLens dataset with added Gaussian context noise and Gaussian prior: parameters set as $\sigma_{n}^{2}=0.1$, $\sigma_{\gamma }^{2}=0.6$.

**Synthetic Datasets**: For synthetic datasets, we go beyond Gaussian bandits and evaluate our algorithms for logistic contextual bandits (see Figure 1 (Left) and (Center)). In both these settings, we consider Gaussian contexts and context noise as in Section 3.1 with parameters $\Sigma_{c}=I$, $\Sigma_{\gamma }=\sigma_{\gamma }^{2}I$, $\Sigma_{n}=\sigma_{n}^{2}I$ for some $\sigma_{\gamma }^{2},\sigma_{n}^{2}>0$. We further consider action a∈A and context c∈C to be d=5 dimensional vectors with $a_{i}$ and $c_{i}$, respectively, denoting their *i*th component. We use $\phi \left(a,c\right)=[a_{1}^{2},a_{2}^{2},a_{3}^{2},a_{4}^{2},a_{5}^{2},c_{1}^{2},c_{2}^{2},c_{3}^{2},c_{4}^{2},c_{5}^{2},a_{1}c_{1},a_{2}c_{2},a_{3}c_{3},a_{4}c_{4},$$a_{5}c_{5}]$ as the m=15 dimensional feature vector.

**Gaussian Bandits**: The mean reward function is given by $f\left(\theta^{*},a,c\right)=\phi {\left(a,c\right)}^{⊤}\theta^{*}$ with the feature map described above. Other parameters are fixed as $\sigma_{\gamma }^{2}=\sigma_{n}^{2}=1.1$, $\sigma^{2}=2$ and λ=0.01. Plots are averaged over 100 independent trials.

**Logistic Bandits**: The reward $r_{t}\in \left\{0,1\right\}$ is Bernoulli with mean reward given by $\mu (\phi {\left(a,c\right)}^{⊤}\theta^{*})$, where μ(z)=1/(1+exp(−z)) is the sigmoid function. We consider a Gaussian prior N(0,I) over $\theta^{*}$. In Algorithm 1, we choose the sampling distribution

$${\bar{P}}_{t}\left(\theta^{*}\right)\propto P\left(\theta^{*}\right)\prod_{\tau =1}^{t-1}\mathrm{Ber}\left(\mu \left(\psi {\left(a_{\tau },{\hat{c}}_{\tau }|H_{\hat{c}}\right)}^{⊤}\theta^{*}\right)\right).$$

However, the posterior ${\bar{P}}_{t}\left(\theta^{*}\right)$ is analytically intractable since Bernoulli reward-Gaussian prior forms a non-conjugate distribution pair. Consequently, we use Langevin Monte Carlo (LMC) [20] to sample from ${\bar{P}}_{t}\left(\theta^{*}\right)$. We run LMC for I=50 iterations with learning rate $\eta_{t}=0.2/t$ and inverse temperature $\beta^{-1}=0.001$. Plots are averaged over 10 independent trials.

***MovieLens Dataset***: We use the MovieLens-100K dataset [21] to evaluate the performances. To utilise this dataset, we first perform non-negative matrix factorization on the rating matrix $R=\left[r_{c,a}\right]\in R^{943\times 1682}$ with 3 latent factors to obtain R=WH, where $W\in R^{943\times 3}$ and $H\in R^{3\times 1682}$. Each row vector $W_{c}$ corresponds to an user context, while each column vector $H_{a}$ corresponds to movie (action) features. The mean and variance of the Gaussian context distribution is estimated from the row vectors of *W*. We then add Gaussian noise to context as in the synthetic settings with $\sigma_{n}^{2}=0.1$.

We apply K-means algorithm to the column vectors of *H* to group the actions into K=20 clusters. We use $m_{k}\in R^{3}$ to denote the centroid and $v_{k}$ to denote the variance of the *k*th cluster. We then fix the mean and variance of the Gaussian prior over $\theta^{*}$ as $\mu_{\theta }=\left(m_{1},\ldots ,m_{K}\right)$ and $\Sigma_{\theta }=\mathrm{diag}\left(v_{1}I_{3},\ldots ,v_{K}I_{3}\right)$, with $I_{3}$ denoting the 3×3 identity matrix, respectively. The feature vector ϕ(a,c) is then fixed as a 60-dimensional vector with vector $W_{c}$ at the index of the cluster *k* to which action *a* belongs and zeros everywhere else. We further add mean-zero Gaussian noise to the mean reward $\phi {\left(a,c\right)}^{⊤}\theta^{*}$ with variance $\sigma^{2}=0.01$. The Bayesian oracle in this experiment has access to the exact context noise parameter $\gamma^{*}$ sampled from the Gaussian prior with variance $\Sigma_{\gamma }=\sigma_{\gamma }^{2}I$, as well as the true $\theta^{*}$ sampled from the Gaussian prior $P(\theta^{*})$.

***Baselines***: We compare our algorithms with two baselines: TS_naive and TS_oracle. In TS_naive, the agent observes only noisy contexts but is unaware of the presence of noise. Consequently, it naively implements conventional TS with noisy context ${\hat{c}}_{t}$. This sets the benchmark for the worst-case achievable regret. The second baseline TS_oracle assumes that the agent knows the true channel parameter $\gamma^{*}$, a setting studied in [18], and can thus perform exact denoising via the predictive posterior distribution $P(c_{t}|\hat{c},\gamma^{*})$. This algorithm sets the benchmark for the best achievable regret.

Figure 1 (Left) corroborates our theoretical findings for Gaussian bandits. In particular, our algorithms (Algorithms 1 and  2) demonstrate sub-linear regret and achieve robust performance comparable to the best achievable performance of TS_oracle. We remark that while our regret analysis of Gaussian bandits is motivated due to the tractability of posterior distributions and the concentration properties of Gaussians, our empirical results for logistic bandits in Figure 1 (Center) show a promising extension of our algorithms to non-conjugate distributions. Extension of Bayesian regret analysis to such general distributions is left for future work. Further, our experiments on MovieLens data in Figure 1 (Right) validate the effectiveness of our algorithm in comparison to the benchmarks. The plot shows that our approach outperforms TS_naive and achieves comparable regret as that of TS_oracle which is the best achievable regret.

## 6. Conclusions

We studied a stochastic CB problem where the agent observes noisy contexts through a noise channel with an unknown channel parameter. For Gaussian bandits and Gaussian context noise, we introduced a TS algorithm that achieves $\tilde{O}\left(m\sqrt{T}\right)$ Bayesian regret. The setting of Gaussian bandits with Gaussian noise was chosen for easy tractability of posterior distributions used in the proposed TS algorithms. We believe that the algorithm and key lemmas can be extended to when the likelihood-prior form conjugate distributions. Extension to general distributions is left for future work.

Finally, we conjecture that our proposed modified TS algorithm and the information-theoretic Bayesian regret analysis could be extended to noisy contexts in multi-task bandit settings. In this regard, a good starting point would be to leverage prior works that study multi-armed hierarchical bandits [22] and contextual hierarchical bandits [23] with linear-Gaussian reward models. However, the critical challenge is to evaluate the posterior mismatch which requires a case-by-case analysis.

## Figures and Tables

**Table 1:** Comparison of the regret bounds of our proposed TS algorithm for noisy CB with state-of-the art algorithms.

Reference	Setting	Algorithm	Regret	Bound
[8]	Linear CB	LinRel	Frequentist	$\tilde{O}\left(\sqrt{mT}\right)$
[9]	Linear CB	Lin-UCB	Frequentist	$\tilde{O}\left(\sqrt{mT}\right)$
[10]	Linear CB	TS	Frequentist	$O(m\sqrt{T}\log^{3/2}T)$
[17]	Linear CB	TS	Bayesian	$O(m\sqrt{T}\log T)$
[11]	Noisy CB	SampLinUCB	Frequentist	$\tilde{O}\left(m\sqrt{T}\right)$
[12]	Noisy CB	UCB	Frequentist	$\tilde{O}\left(m\sqrt{T}\right)$
[14]	Noisy CB	OFUL	Frequentist	$\tilde{O}\left(m\sqrt{T}\right)$
**Our work**	**Noisy CB**	**TS**	**Bayesian**	** $\tilde{O}\left(m\sqrt{T}\right)$ **

**Figure A1:**
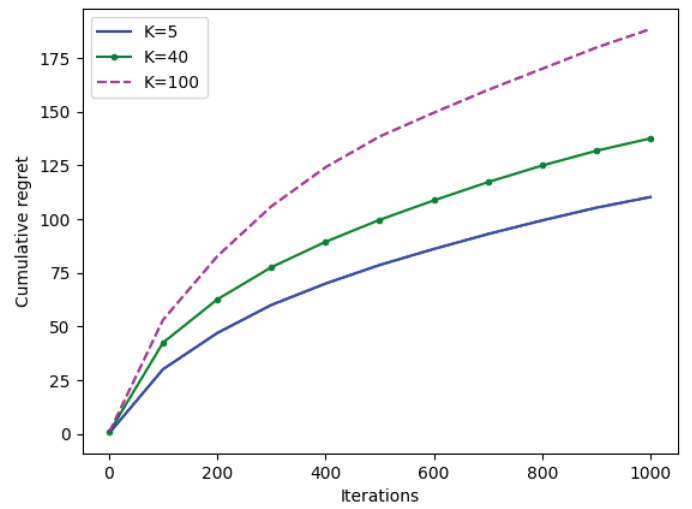
Bayesian cumulative regret of Algorithm 1 as a function of iterations over varying number *K* of actions.

## Data Availability

The data presented in this study are openly available in Kaggle, https://www.kaggle.com/datasets/prajitdatta/movielens-100k-dataset, accessed on 1 July 2023.

## Appendix A. Preliminaries

**Definition** **A1** (Sub-Gaussian Random Variable)**.** *A random variable y is said to be $s^{2}$-sub-Gaussian with respect to the distribution P(y) if the following inequality holds:*

(A1) $$\begin{array}{c} E_{P(y)}[\exp\left(\lambda \left(y-E_{P(y)}\left[y\right]\right)\right]\leq \exp\left(\frac{\lambda^{2}s^{2}}{2}\right). \end{array}$$

**Lemma** **A1** (Change of Measure Inequality)**.** *Let $x\in R^{n}$ be a random vector and $g:R^{n}\to R$ denote a real-valued function. Let P(x) and Q(x) be two probability distributions defined on the space of x. If g(x) is $s^{2}$-sub-Gaussian with respect to Q(x), then the following inequality holds,*

(A2) $$\begin{array}{c} |E_{P(x)}\left[g\left(x\right)\right]-E_{Q(x)}\left[g\left(x\right)\right]|\leq \sqrt{2s^{2}D_{\mathrm{KL}}\left(P\left(x\right)∥Q\left(x\right)\right)}. \end{array}$$

**Proof.** The inequality (A2) follows by using the Donsker-Varadhan inequality (20) with f(x)=λg(x) for λ∈R. This yields that

(A3) $$\begin{array}{cc} D_{\mathrm{KL}}\left(P\left(x\right)∥Q\left(x\right)\right) & \geq E_{P(x)}\left[\lambda g\left(x\right)\right]-\log E_{Q(x)}\left[\exp\left(\lambda g\left(x\right)\right)\right]\\ \; & \geq E_{P(x)}\left[\lambda g\left(x\right)\right]-E_{Q(x)}\left[\lambda g\left(x\right)\right]-\lambda^{2}\frac{s^{2}}{2} \end{array}$$

where the last inequality follows from the assumption of sub-Gaussianity. Rearranging, we obtain

(A4) $$\begin{array}{c} E_{P(x)}\left[\lambda g\left(x\right)\right]-E_{Q(x)}\left[\lambda g\left(x\right)\right]\leq \lambda^{2}\frac{s^{2}}{2}+D_{\mathrm{KL}}\left(P\left(x\right)∥Q\left(x\right)\right). \end{array}$$

For λ>0, we obtain that

(A5) $$\begin{array}{c} E_{P(x)}\left[g\left(x\right)\right]-E_{Q(x)}\left[g\left(x\right)\right]\leq \lambda \frac{s^{2}}{2}+\frac{D_{\mathrm{KL}}\left(P\left(x\right)∥Q\left(x\right)\right)}{\lambda }, \end{array}$$

and optimizing over λ>0 then yields that

(A6) $$\begin{array}{c} E_{P(x)}\left[g\left(x\right)\right]-E_{Q(x)}\left[g\left(x\right)\right]\leq \sqrt{2s^{2}D_{\mathrm{KL}}\left(P\left(x\right)∥Q\left(x\right)\right)}. \end{array}$$

Similarly, for λ<0, we obtain that

(A7) $$\begin{array}{c} E_{Q(x)}\left[g\left(x\right)\right]-E_{P(x)}\left[g\left(x\right)\right]\leq \sqrt{2s^{2}D_{\mathrm{KL}}\left(P\left(x\right)∥Q\left(x\right)\right)}. \end{array}$$

□

**Lemma** **A2.**
*Let $x\in R^{n}$ be distributed according to $Q\left(x\right)=\prod_{i=1}^{n}N\left(x_{i}|\mu_{i},\sigma_{i}^{2}\right)$, i.e., each element of the random vector is independently distributed according to a Gaussian distribution with mean $\mu_{i}$ and variance $\sigma_{i}^{2}$. Let $g\left(x\right)=\max_{i=1,\ldots n}x_{i}$ denote the maximum of n Gaussian random variables. Then, the following inequality holds for λ≥0,*

(A8)
$$\begin{array}{c} \log E_{Q(x)}\left[\exp\left(\lambda g\left(x\right)\right)\right]\leq \log n+\lambda \max_{i}\mu_{i}+\lambda^{2}\frac{\max_{i}\sigma_{i}^{2}}{2}. \end{array}$$

*For any distribution P(x) that is absolutely continuous with respect to Q(x), we then have the following change of measure inequality,*

(A9)
$$\begin{array}{c} E_{P(x)}\left[g\left(x\right)\right]-E_{Q(x)}\left[g\left(x\right)\right]\leq \sqrt{2\left(\log n+D_{\mathrm{KL}}\left(P\left(x\right)∥Q\left(x\right)\right)\right)\max_{i}\sigma_{i}^{2}}. \end{array}$$

**Proof.** The proof of inequality (A8) follows from standard analysis (see [14]). We present it here for the sake of completeness. The following sequence of relations hold for any λ≥0,

(A10) $$\begin{array}{cc} E_{Q(x)}\left[\exp\left(\lambda g\left(x\right)\right)\right]=E_{Q(x)}\left[\max_{i}\exp\left(\lambda x_{i}\right)\right] & \leq \sum_{i=1}^{n}E_{Q(x_{i})}\left[\exp\left(\lambda x_{i}\right)\right]\\ & =\sum_{i=1}^{n}\exp\left(\lambda \mu_{i}+\lambda^{2}\sigma_{i}^{2}/2\right)\\ & \leq n\exp(\lambda \max_{i}\mu_{i}+\lambda^{2}\max_{i}\sigma_{i}^{2}/2). \end{array}$$

Taking logarithm on both sides of the inequality yields the upper bound in (A8). We now apply the DV inequality (20) as in (A3). This yields that

(A11) $$\begin{array}{cc} D_{\mathrm{KL}}\left(P\left(x\right)∥Q\left(x\right)\right) & \geq E_{P(x)}\left[\lambda g\left(x\right)\right]-\log E_{Q(x)}\left[\exp\left(\lambda g\left(x\right)\right)\right]\\ \; & \overset{\left(a\right)}{\geq }E_{P(x)}\left[\lambda g\left(x\right)\right]-\log n-\lambda \max_{i}\mu_{i}-\lambda^{2}\frac{\max_{i}\sigma_{i}^{2}}{2}\\ \; & \overset{\left(b\right)}{\geq }E_{P(x)}\left[\lambda g\left(x\right)\right]-E_{Q(x)}\left[\lambda g\left(x\right)\right]-\log n-\lambda^{2}\frac{\max_{i}\sigma_{i}^{2}}{2}, \end{array}$$

where the inequality in (a) follows from (A8). The inequality in (b) follows from observing that $\max_{i}x_{i}\geq x_{i}$ for all *i*, whereby we obtain that $E_{Q(x)}\left[\max_{i}x_{i}\right]\geq \mu_{i}$ which holds for all *i*. The latter inequality implies that $E_{Q(x)}\left[\max_{i}x_{i}\right]\geq \max_{i}\mu_{i}$. Re-arranging and optimizing over λ≥0 then yields the required inequality in (A9). □

## Appendix B. Linear-Gaussian Contextual Bandits with Delayed Contexts

In this section, we provide all the details relevant to the Bayesian cumulative regret analysis of TS for delayed, linear-Gaussian contextual bandits.

### Appendix B.1. TS Algorithm for Linear-Gaussian Bandits with Delayed True Contexts

The pseudocode for the TS algorithm for Gaussian bandits is given in Algorithm A1.

**Algorithm A1**: TS with Delayed Contexts for Gaussian Bandits ($\pi_{\mathrm{delay}}^{\mathrm{TS}}$)
1: Given parameters: $\left(\Sigma_{n},\sigma^{2},\lambda ,\Sigma_{\gamma },\mu_{c},\Sigma_{c}\right)$. Initialize ${\tilde{\mu }}_{0}=0\in R^{m}$ and ${\tilde{\Sigma }}_{0}^{-1}=\left(1/\lambda \right)I$ 2: **for** t=1,⋯,T **do** 3: The environment selects a true context $c_{t}$. 4: Agent observes noisy context ${\hat{c}}_{t}$. 5: Agent computes ${\tilde{R}}_{t}$ and ${\tilde{V}}_{t}$ using (29) and (30) to evaluate $P\left(c_{t}|{\hat{c}}_{t},H_{t-1,c,\hat{c}}\right)=N\left({\tilde{V}}_{t},{\tilde{R}}_{t}^{-1}\right)$. 6: Agent samples $\theta_{t}∼N\left({\tilde{\mu }}_{t-1},{\tilde{\Sigma }}_{t-1}^{-1}\right)$ where ${\tilde{\mu }}_{t-1}$ and ${\tilde{\Sigma }}_{t-1}$ are defined as in (32) and (31). 7: Agent chooses action $a_{t}$ as in (33) using $\theta_{t}$ and $P(c_{t}|{\hat{c}}_{t},H_{t-1,c,\hat{c}})$. 8: Agent observes reward $r_{t}$ corresponding to $a_{t}$, and the true context $c_{t}$. 9: **end for**

### Appendix B.2. Derivation of Posterior and Predictive Posterior Distributions

In this section, we provide detailed derivation of posterior predictive distribution for Gaussian bandits. To this end, we first derive the exact predictive distribution $P(c_{t}|{\hat{c}}_{t},\gamma^{*})$.

#### Appendix B.2.1. Derivation of $P(c_{t}|{\hat{c}}_{t},\gamma^{*})$

We begin by noting that

$$\begin{array}{cc} P\left(c_{t}|{\hat{c}}_{t},\gamma^{*}\right)=\frac{P\left(c_{t}\right)P\left({\hat{c}}_{t}|c_{t},\gamma^{*}\right)}{P({\hat{c}}_{t}|\gamma^{*})} & \propto P\left(c_{t}\right)P\left({\hat{c}}_{t}|c_{t},\gamma^{*}\right)\\ \; & =N\left(\mu_{c},\Sigma_{c}\right)N\left(c_{t}+\gamma^{*},\Sigma_{n}\right). \end{array}$$

Subsequently,

(A12) $$\begin{array}{cc} \log(P\left(c_{t}|{\hat{c}}_{t},\gamma^{*}\right)) & \propto \log(P\left(c_{t}\right)P\left({\hat{c}}_{t}|c_{t},\gamma^{*}\right))\\ & \propto -\frac{1}{2}\left({\left(c_{t}-\mu_{c}\right)}^{⊤}\Sigma_{c}^{-1}\left(c_{t}-\mu \right)+{\left({\hat{c}}_{t}-c_{t}-\gamma^{*}\right)}^{⊤}\Sigma_{n}^{-1}\left({\hat{c}}_{t}-c_{t}-\gamma^{*}\right)\right)\\ \; & =-\frac{1}{2}(c_{t}^{⊤}\left(\Sigma_{c}^{-1}+\Sigma_{n}^{-1}\right)c_{t}-\left(\mu_{c}^{⊤}\Sigma_{c}^{-1}+{\left({\hat{c}}_{t}-\gamma^{*}\right)}^{⊤}\Sigma_{n}^{-1}\right)c_{t}\\ \; & -c_{t}^{⊤}\left(\Sigma_{c}^{-1}\mu_{c}+\Sigma_{n}^{-1}\left({\hat{c}}_{t}-\gamma^{*}\right)\right)+{\left({\hat{c}}_{t}-\gamma^{*}\right)}^{⊤}\Sigma_{n}^{-1}\left({\hat{c}}_{t}-\gamma^{*}\right))\\ \; & =-\frac{1}{2}(c_{t}^{⊤}Mc_{t}-A_{t}^{⊤}Mc_{t}-c_{t}^{⊤}MA+A_{t}^{⊤}MA-A_{t}^{⊤}MA\\ \; & +{\left({\hat{c}}_{t}-\gamma^{*}\right)}^{⊤}\Sigma_{n}^{-1}\left({\hat{c}}_{t}-\gamma^{*}\right)), \end{array}$$

where we have defined

(A13) $$\begin{array}{cc} M & =\Sigma_{c}^{-1}+\Sigma_{n}^{-1} \end{array}$$

(A14) $$\begin{array}{cc} A_{t} & ={\left(M^{-1}\right)}^{⊤}\left(\Sigma_{c}^{-1}\mu_{c}+\Sigma_{n}^{-1}\left({\hat{c}}_{t}-\gamma^{*}\right)\right). \end{array}$$

From (A12), we obtain

$$\begin{array}{c} \log\left(P\left(c_{t}|{\hat{c}}_{t},\gamma^{*}\right)\right)\propto -\frac{1}{2}\left(c_{t}^{⊤}Mc_{t}-A_{t}^{⊤}Mc_{t}-c_{t}^{⊤}MA+A_{t}^{⊤}MA\right). \end{array}$$

This implies that

(A15) $$\begin{array}{cc} P(c_{t}|{\hat{c}}_{t},\gamma^{*}) & =N(A_{t},M^{-1}). \end{array}$$

#### Appendix B.2.2. Derivation of $P({\hat{c}}_{t}|\gamma^{*})$

We now derive the distribution $P({\hat{c}}_{t}|\gamma^{*})$ which is defined in (3) as

$$\begin{array}{c} P\left({\hat{c}}_{t}|\gamma^{*}\right)=E_{P(c_{t})}\left[P\left({\hat{c}}_{t}|c_{t},\gamma^{*}\right)\right]. \end{array}$$

Hence, $P({\hat{c}}_{t}|\gamma^{*})$ can be obtained by marginalizing the joint distribution $P\left(c_{t}\right)P\left({\hat{c}}_{t}|c_{t},\gamma^{*}\right)=P\left(c_{t}|{\hat{c}}_{t},\gamma^{*}\right)P\left({\hat{c}}_{t}|\gamma^{*}\right)$ over $c_{t}$. To this end, we use (A12) to obtain,

$$\begin{array}{cc} \log(P\left(c_{t}\right)P\left({\hat{c}}_{t}|c_{t},\gamma^{*}\right)) & =\log(P\left(c_{t}|{\hat{c}}_{t},\gamma^{*}\right)P\left({\hat{c}}_{t}|\gamma^{*}\right))\\ & \propto \log\left(P\left(c_{t}|{\hat{c}}_{t},\gamma^{*}\right)\right)-\frac{1}{2}\left(-A_{t}^{⊤}MA+{\left({\hat{c}}_{t}-\gamma^{*}\right)}^{⊤}\Sigma_{n}^{-1}\left({\hat{c}}_{t}-\gamma^{*}\right)\right), \end{array}$$

which implies that

$$\begin{array}{cc} \log(P\left({\hat{c}}_{t}|\gamma^{*}\right)) & \propto -\frac{1}{2}\left(-A_{t}^{⊤}MA+{\left({\hat{c}}_{t}-\gamma^{*}\right)}^{⊤}\Sigma_{n}^{-1}\left({\hat{c}}_{t}-\gamma^{*}\right)\right)\\ \; & \propto -\frac{1}{2}({\hat{c}}_{t}^{⊤}\left(\Sigma_{n}^{-1}-\Sigma_{n}^{-1}{\left(M^{-1}\right)}^{⊤}\Sigma_{n}^{-1}\right){\hat{c}}_{t}-{\hat{c}}_{t}^{⊤}(\Sigma_{n}^{-1}{\left(M^{-1}\right)}^{⊤}\left(-\Sigma_{n}^{-1}\gamma^{*}+\Sigma_{c}^{-1}\mu_{c}\right)\\ & +\Sigma_{n}^{-1}\gamma^{*})-\left(\left(\mu_{c}^{⊤}\Sigma_{c}^{-1}-\gamma^{*⊤}\Sigma_{n}^{-1}\right)\left(M^{-1}\right)\Sigma_{n}^{-1}+\gamma^{*⊤}\Sigma_{n}^{-1}\right)\hat{c})\\ \; & =-\frac{1}{2}\left({\hat{c}}_{t}^{⊤}G{\hat{c}}_{t}-F^{⊤}G{\hat{c}}_{t}-G^{⊤}F{\hat{c}}_{t}\right)\\ & \propto -\frac{1}{2}\left({\left({\hat{c}}_{t}-F\right)}^{⊤}G\left({\hat{c}}_{t}-F\right)\right), \end{array}$$

where

(A16) $$\begin{array}{cc} G & =\Sigma_{n}^{-1}-\Sigma_{n}^{-1}{\left(M^{-1}\right)}^{⊤}\Sigma_{n}^{-1} \end{array}$$

(A17) $$\begin{array}{cc} F & ={\left(G^{-1}\right)}^{⊤}\left(\Sigma_{n}^{-1}{\left(M^{-1}\right)}^{⊤}\left(-\Sigma_{n}^{-1}\gamma^{*}+\Sigma_{c}^{-1}\mu_{c}\right)+\Sigma_{n}^{-1}\gamma^{*}\right)={\left(G^{-1}\right)}^{⊤}\left(G\gamma^{*}+\Sigma_{n}^{-1}{\left(M^{-1}\right)}^{⊤}\Sigma_{c}^{-1}\mu_{c}\right). \end{array}$$

Thus,

(A18) $$\begin{array}{c} P\left({\hat{c}}_{t}|\gamma^{*}\right)=N\left(F,G^{-1}\right). \end{array}$$

#### Appendix B.2.3. Derivation of $P(\gamma^{*}|H_{t-1,c,\hat{c}})$

We now derive the posterior distribution $P(\gamma^{*}|H_{t-1,c,\hat{c}})$. To this end, we use Baye’s theorem as

$$\begin{array}{cc} P(\gamma^{*}|H_{t-1,c,\hat{c}}) & \propto \prod_{\tau =1}^{t-1}P\left({\hat{c}}_{\tau }|c_{\tau },\gamma^{*}\right)P\left(\gamma^{*}\right)\\ \; & =\prod_{\tau =1}^{t-1}N\left(c_{\tau }+\gamma^{*},\Sigma_{n}\right)N\left(0,\Sigma_{\gamma }\right). \end{array}$$

We then have,

$$\begin{array}{cc} \log P(\gamma^{*}|H_{t-1,c,\hat{c}}) & \propto -\frac{1}{2}\left(\sum_{\tau =1}^{t-1}\left({\left({\hat{c}}_{\tau }-c_{\tau }-\gamma^{*}\right)}^{⊤}\Sigma_{n}^{-1}\left({\hat{c}}_{\tau }-c_{\tau }-\gamma^{*}\right)\right)+\gamma^{*⊤}\Sigma_{\gamma }^{-1}\gamma^{*}\right)\\ \; & =-\frac{1}{2}\left(\sum_{\tau =1}^{t-1}\left({\left(-{\hat{c}}_{\tau }+c_{\tau }+\gamma^{*}\right)}^{⊤}\Sigma_{n}^{-1}\left(-{\hat{c}}_{\tau }+c_{\tau }+\gamma^{*}\right)\right)+\gamma^{*⊤}\Sigma_{\gamma }^{-1}\gamma^{*}\right)\\ \; & \propto -\frac{1}{2}(\gamma^{*⊤}\left(\left(t-1\right)\Sigma_{n}^{-1}+\Sigma_{\gamma }^{-1}\right)\gamma^{*}-\gamma^{*⊤}\Sigma_{n}^{-1}\left(\sum_{\tau =1}^{t-1}\left({\hat{c}}_{\tau }-c_{\tau }\right)\right)\\ & -{\left(\sum_{\tau =1}^{t-1}\left({\hat{c}}_{\tau }-c_{\tau }\right)\right)}^{⊤}\Sigma_{n}^{-1}\gamma^{*}). \end{array}$$

Consequently, we obtain that,

(A19) $$\begin{array}{c} P\left(\gamma^{*}|H_{t-1,c,\hat{c}}\right)=N\left(\gamma^{*}|Y_{t},W_{t}^{-1}\right) \end{array}$$

where

(A20) $$\begin{array}{cc} W_{t} & =\left(t-1\right)\Sigma_{n}^{-1}+\Sigma_{\gamma }^{-1} \end{array}$$

(A21) $$\begin{array}{cc} Y_{t} & ={\left(W_{t}^{-1}\right)}^{⊤}\Sigma_{n}^{-1}\sum_{\tau =1}^{t-1}\left({\hat{c}}_{\tau }-c_{\tau }\right). \end{array}$$

#### Appendix B.2.4. Derivation of Posterior Predictive Distribution $P(c_{t}|{\hat{c}}_{t},H_{t-1,c,\hat{c}})$

Using results from previous subsections, we are now ready to derive the posterior predictive distribution $P(c_{t}|{\hat{c}}_{t},H_{t-1,c,\hat{c}})$. Note, that $P\left(c_{t}|{\hat{c}}_{t},H_{t-1,c,\hat{c}}\right)=E_{P(\gamma^{*}|H_{t-1,c,\hat{c}})}\left[P\left(c_{t}|{\hat{c}}_{t},\gamma^{*}\right)\right]$. We then have the following set of relations:

$$\begin{array}{cc} \; & \log\left(P\left(\gamma^{*}|H_{t-1,c,\hat{c}}\right)P\left(c_{t}|{\hat{c}}_{t},\gamma^{*}\right)\right)\\ & \propto -\frac{1}{2}\left({\left(c_{t}-A_{t}\right)}^{⊤}M\left(c_{t}-A_{t}\right)+{\left(\gamma^{*}-Y_{t}\right)}^{⊤}W_{t}\left(\gamma^{*}-Y_{t}\right)\right)\\ \; & =-\frac{1}{2}({\left(c_{t}-D-E_{t}+{\left(M^{-1}\right)}^{⊤}\Sigma_{n}^{-1}\gamma^{*}\right)}^{⊤}M\left(c_{t}-D-E_{t}+{\left(M^{-1}\right)}^{⊤}\Sigma_{n}^{-1}\gamma^{*}\right)\\ & +{\left(\gamma^{*}-Y_{t}\right)}^{⊤}W_{t}\left(\gamma^{*}-Y_{t}\right))\\ \; & \propto -\frac{1}{2}(\gamma^{*⊤}\left(\Sigma_{n}^{-1}{\left(M^{-1}\right)}^{⊤}\Sigma_{n}^{-1}+W_{t}\right)\gamma^{*}-\gamma^{*⊤}\left(\Sigma_{n}^{-1}\left(D+E_{t}-c_{t}\right)+W_{t}Y_{t}\right)-({\left(D+E_{t}-c_{t}\right)}^{⊤}\Sigma_{n}^{-1}\\ & +Y_{t}^{⊤}W_{t})\gamma^{*}+{\left(c_{t}-D-E_{t}\right)}^{⊤}M\left(c_{t}-D-E_{t}\right))\\ \; & =-\frac{1}{2}\left(\gamma^{*⊤}{\tilde{H}}_{t}\gamma^{*}-\gamma^{*⊤}{\tilde{H}}_{t}^{⊤}{\tilde{J}}_{t}-{\tilde{J}}_{t}^{⊤}{\tilde{H}}_{t}\gamma^{*}+{\tilde{J}}_{t}^{⊤}{\tilde{H}}_{t}{\tilde{J}}_{t}-{\tilde{J}}_{t}^{⊤}{\tilde{H}}_{t}{\tilde{J}}_{t}+{\left(c_{t}-D-E_{t}\right)}^{⊤}M\left(c_{t}-D-E_{t}\right)\right)\\ \; & \propto \log\left(P\left(\gamma^{*}|H_{t,c,\hat{c}}\right)\right)-\frac{1}{2}\left(-{\tilde{J}}_{t}^{⊤}{\tilde{H}}_{t}{\tilde{J}}_{t}+{\left(c_{t}-D-E_{t}\right)}^{⊤}M\left(c_{t}-D-E_{t}\right)\right) \end{array}$$

where $D={\left(M^{-1}\right)}^{⊤}\Sigma_{c}^{-1}\mu_{c}$, $E_{t}={\left(M^{-1}\right)}^{⊤}\Sigma_{n}^{-1}{\hat{c}}_{t}$, ${\tilde{H}}_{t}=\Sigma_{n}^{-1}{\left(M^{-1}\right)}^{⊤}\Sigma_{n}^{-1}+W_{t}$ and ${\tilde{J}}_{t}={\left({\tilde{H}}_{t}^{-1}\right)}^{⊤}\left(\Sigma_{n}^{-1}\left(D+E_{t}-c_{t}\right)+W_{t}Y_{t}\right)$.

Since $\log\left(P\left(\gamma^{*}|H_{t-1,c,\hat{c}}\right)P\left(c_{t}|{\hat{c}}_{t},\gamma^{*}\right)\right)=\log\left(P\left(c_{t}|{\hat{c}}_{t},H_{t-1,c,\hat{c}}\right)P\left(\gamma^{*}|H_{t,c,\hat{c}}\right)\right)$, we obtain

$$\begin{array}{cc} \log(P\left(c_{t}|{\hat{c}}_{t},H_{t-1,c,\hat{c}}\right)) & \propto -\frac{1}{2}\left(-{\tilde{J}}_{t}^{⊤}{\tilde{H}}_{t}{\tilde{J}}_{t}+{\left(c_{t}-D-E_{t}\right)}^{⊤}M\left(c_{t}-D-E_{t}\right)\right)\\ \; & \propto -\frac{1}{2}(c_{t}^{⊤}\left(M-\Sigma_{n}^{-1}{\left({\tilde{H}}_{t}^{-1}\right)}^{⊤}\Sigma_{n}^{-1}\right)c_{t}-c_{t}^{⊤}(M\left(D+E_{t}\right)\\ \; & -\Sigma_{n}^{-1}{\left({\tilde{H}}_{t}^{-1}\right)}^{⊤}\left(\Sigma_{n}^{-1}\left(D+E_{t}\right)+W_{t}Y_{t}\right))-(M\left(D+E_{t}\right)\\ & -\Sigma_{n}^{-1}{\left({\tilde{H}}_{t}^{-1}\right)}^{⊤}\left(\Sigma_{n}^{-1}\left(D+E_{t}\right)+W_{t}Y_{t}\right){)}^{⊤}c_{t}). \end{array}$$

This gives

(A22) $$\begin{array}{cc} P(c_{t}|{\hat{c}}_{t},H_{t-1,c,\hat{c}}) & =N({\tilde{V}}_{t},{\tilde{R}}_{t}^{-1})\;where \end{array}$$

(A23) $$\begin{array}{cc} {\tilde{R}}_{t} & =M-\Sigma_{n}^{-1}{\left({\tilde{H}}_{t}^{-1}\right)}^{⊤}\Sigma_{n}^{-1} \end{array}$$

(A24) $$\begin{array}{cc} {\tilde{V}}_{t} & ={\left({\tilde{R}}_{t}^{-1}\right)}^{⊤}\left(M\left(D+E_{t}\right)-\Sigma_{n}^{-1}{\left({\tilde{H}}_{t}^{-1}\right)}^{⊤}\left(\Sigma_{n}^{-1}\left(D+E_{t}\right)+W_{t}Y_{t}\right)\right). \end{array}$$

### Appendix B.3. Proof of Lemma 3

We now present the proof of Lemma 3. To this end, we first recall that $E_{t}\left[\cdot \right]=E\left[\cdot |F_{t}\right]$ where $F_{t}=H_{t-1,r,a,c,\hat{c}}\cup {\hat{c}}_{t}$, and we denote $P_{t}\left(\theta^{*}\right)$ as the posterior distribution $P(\theta^{*}|H_{t-1,r,a,c})$ of $\theta^{*}$ given the history of observed reward-action-context tuples. We can then equivalently write $R_{d,\mathrm{CB}}^{T}$ as

(A25) $$\begin{array}{cc} R_{d,\mathrm{CB}}^{T} & =\sum_{t=1}^{T}E\left[E_{t}\left[\psi {\left({\hat{a}}_{t},{\hat{c}}_{t}|H_{c,\hat{c}}\right)}^{⊤}\theta^{*}-\psi {\left(a_{t},{\hat{c}}_{t}|H_{c,\hat{c}}\right)}^{⊤}\theta^{*}\right]\right]\\ \; & =\sum_{t=1}^{T}E[E_{t}\underset{:=\Delta_{t}}{\underset{︸}{\left[f\left(\theta^{*},{\hat{a}}_{t},c_{t}\right)-f\left(\theta^{*},a_{t},c_{t}\right)\right]}}], \end{array}$$

where $\psi \left(a,{\hat{c}}_{t}|H_{c,\hat{c}}\right)=E_{P(c_{t}|{\hat{c}}_{t},H_{t-1,c,\hat{c}})}\left[\phi \left(a,c_{t}\right)\right]$ is as defined in (33) and we have used $f\left(\theta^{*},a_{t},c_{t}\right)=\phi {\left(a_{t},c_{t}\right)}^{⊤}\theta^{*}$ to denote the mean-reward function. To obtain an upper bound on $R_{d,\mathrm{CB}}^{T}$, we define the following *lifted information ratio* as in [19],

(A26) $$\begin{array}{c} \Gamma_{t}=\frac{{\left(E_{t}\left[\Delta_{t}\right]\right)}^{2}}{\Lambda_{t}} \end{array}$$

where

(A27) $$\begin{array}{c} \Lambda_{t}=E_{t}\left[{\left(f\left(\theta^{*},a_{t},c_{t}\right)-\bar{f}\left(a_{t},c_{t}\right)\right)}^{2}\right], \end{array}$$

with $\bar{f}\left(a_{t},c_{t}\right)=E_{t}\left[f\left(\theta^{*},a_{t},c_{t}\right)|a_{t},c_{t}\right]$ denoting the expectation of mean reward with respect to the posterior distribution $P_{t}\left(\theta^{*}\right)$. Subsequently, we obtain the following upper bound

(A28) $$\begin{array}{c} R_{d,\mathrm{CB}}^{T}\leq \sum_{t=1}^{T}E\left[\sqrt{\Gamma_{t}\Lambda_{t}}\right]\leq \sqrt{E\left[\sum_{t=1}^{T}\Gamma_{t}\right]\left[\sum_{t=1}^{T}E\left[\Lambda_{t}\right]\right]}, \end{array}$$

where the last inequality follows by an application of Cauchy–Schwarz inequality. An upper bound on $R_{d,\mathrm{CB}}^{T}$ then follows by obtaining an upper bound on the lifted information ratio $\Gamma_{t}$ as well as on $\Lambda_{t}.$

We first evaluate the term $\Lambda_{t}$. To this end, note that $\bar{f}\left(a_{t},c_{t}\right)=\phi {\left(a_{t},c_{t}\right)}^{⊤}{\tilde{\mu }}_{t-1}$, with ${\tilde{\mu }}_{t-1}$ defined as in (32). Using this, we obtain

$$\begin{array}{cc} \Lambda_{t} & =E_{t}\left[{\left(\phi {\left(a_{t},c_{t}\right)}^{⊤}\left(\theta^{*}-{\tilde{\mu }}_{t-1}\right)\right)}^{2}\right] \end{array}$$

(A29) $$\begin{array}{cc} \; & =E_{t}\left[\phi {\left(a_{t},c_{t}\right)}^{⊤}\left(\theta^{*}-{\tilde{\mu }}_{t-1}\right){\left(\theta^{*}-{\tilde{\mu }}_{t-1}\right)}^{⊤}\phi \left(a_{t},c_{t}\right)\right] \end{array}$$

(A30) $$\begin{array}{cc} \; & =E_{t}\left[\phi {\left(a_{t},c_{t}\right)}^{⊤}{\tilde{\Sigma }}_{t-1}^{-1}\phi \left(a_{t},c_{t}\right)\right]=E_{t}\left[∥\phi \left(a_{t},c_{t}\right){∥}_{{\tilde{\Sigma }}_{t-1}^{-1}}^{2}\right] \end{array}$$

where ${\tilde{\Sigma }}_{t-1}$ is as in (31), and the third equality follows since conditional on $F_{t}$, $\left(a_{t},c_{t}\right)$ is independent of $\theta^{*}$. Subsequently, we can apply the elliptical potential lemma Lemma 19.4 in [24] using the assumption that ${∥\phi \left(\cdot ,\cdot \right)∥}_{2}\leq 1$ and that $\sigma^{2}/\lambda \geq 1$. This results in

(A31) $$\begin{array}{cc} \sum_{t=1}^{T}{∥\phi \left(a_{t},c_{t}\right)∥}_{{\tilde{\Sigma }}_{t-1}^{-1}}^{2} & =\sigma^{2}\sum_{t=1}^{T}{∥\phi \left(a_{t},c_{t}\right)∥}_{{\left(\sigma^{2}{\tilde{\Sigma }}_{t-1}\right)}^{-1}}^{2}\\ & \leq 2\sigma^{2}\log\frac{\det({\tilde{\Sigma }}_{T})}{\det(\sigma^{2}/\lambda I)}=2\sigma^{2}\left(m\log\left(\sigma^{2}/\lambda +T/m\right)-m\log\left(\sigma^{2}/\lambda \right)\right)\\ & =2m\sigma^{2}\log\left(1+\frac{T\lambda }{m\sigma^{2}}\right). \end{array}$$

To upper bound the lifted information ratio term $\Gamma_{t}$, we can use Lemma 7 in [19]. To demonstrate how to leverage results from [19], we start by showing that the inequality $\Gamma_{t}\leq m$ holds. To this end, we note that the lifted information ratio can be equivalently written as

(A32) $$\begin{array}{c} \Gamma_{t}=\frac{{\left(E_{t}\left[f\left(\theta_{t},a_{t},c_{t}\right)-\bar{f}\left(a_{t},c_{t}\right)\right]\right)}^{2}}{\Lambda_{t}} \end{array}$$

which follows since

(A33) $$\begin{array}{cc} E_{t}\left[\Delta_{t}\right] & =E_{t}\left[f\left(\theta^{*},{\hat{a}}_{t},c_{t}\right)-f\left(\theta^{*},a_{t},c_{t}\right)\right]\\ \; & =E_{t}\left[f\left(\theta^{*},{\hat{a}}_{t},c_{t}\right)-\bar{f}\left(a_{t},c_{t}\right)\right]\\ \; & =\sum_{a^{\prime }}P_{t}\left({\hat{a}}_{t}=a^{\prime }\right)E_{t}\left[f\left(\theta^{*},a^{\prime },c_{t}\right)|{\hat{a}}_{t}=a^{\prime }\right]-\sum_{a^{\prime }}P_{t}\left(a_{t}=a^{\prime }\right)E_{t}\left[\bar{f}\left(a_{t}=a^{\prime },c_{t}\right)\right]\\ \; & =\sum_{a^{\prime }}P_{t}\left({\hat{a}}_{t}=a^{\prime }\right)\left(E_{t}\left[f\left(\theta^{*},a^{\prime },c_{t}\right)|{\hat{a}}_{t}=a^{\prime }\right]-E_{t}\left[\bar{f}\left(a^{\prime },c_{t}\right)\right]\right), \end{array}$$

where the second equality holds since conditioned on $F_{t}$, $\left(a_{t},c_{t}\right)$ and $\theta^{*}$ are independent. In the third equality, we denote $P_{t}\left({\hat{a}}_{t}\right)=P\left({\hat{a}}_{t}|F_{t}\right)$ and $P_{t}\left(a_{t}\right)=P\left(a_{t}|F_{t}\right)$. Using these, the last equality follows since $a_{t}\overset{d}{=}{\hat{a}}_{t}$, i.e, $P_{t}\left(a_{t}\right)=P_{t}\left({\hat{a}}_{t}\right)$. Now, let us define a K×K matrix *M* with entries given by

(A34) $$\begin{array}{c} M_{a,a^{\prime }}=\sqrt{P_{t}\left({\hat{a}}_{t}=a^{\prime }\right)P_{t}\left(a_{t}=a\right)}\left(E_{t}\left[f\left(\theta^{*},a,c_{t}\right)|{\hat{a}}_{t}=a^{\prime }\right]-E_{t}\left[\bar{f}\left(a,c_{t}\right)\right]\right). \end{array}$$

Using this and noting that $P_{t}\left({\hat{a}}_{t}\right)=P_{t}\left(a_{t}\right)$, we obtain that $E_{t}\left[\Delta_{t}\right]=\mathrm{Tr}\left(M\right)$. We now try to bound $\Lambda_{t}$ in terms of the matrix *M*. To see this, we can equivalently write $\Lambda_{t}$ as

(A35) $$\begin{array}{cc} \Lambda_{t} & =\sum_{a}P_{t}\left(a_{t}=a\right)E_{t}\left[{\left(f\left(\theta^{*},a,c_{t}\right)-\bar{f}\left(a,c_{t}\right)\right)}^{2}\right]\\ \; & =\sum_{a,a^{\prime }}P_{t}\left(a_{t}=a\right)P_{t}\left({\hat{a}}_{t}=a^{\prime }\right)E_{t}\left[{\left(f\left(\theta^{*},a,c_{t}\right)-\bar{f}\left(a,c_{t}\right)\right)}^{2}|{\hat{a}}_{t}=a^{\prime }\right]\\ \; & \geq \sum_{a,a^{\prime }}P_{t}\left(a_{t}=a\right)P_{t}\left({\hat{a}}_{t}=a^{\prime }\right){\left(E_{t}\left[f\left(\theta^{*},a,c_{t}\right)-\bar{f}\left(a,c_{t}\right)|{\hat{a}}_{t}=a^{\prime }\right]\right)}^{2}\\ \; & =\sum_{a,a^{\prime }}P_{t}\left(a_{t}=a\right)P_{t}\left({\hat{a}}_{t}=a^{\prime }\right){\left(E_{t}\left[f\left(\theta^{*},a,c_{t}\right)|{\hat{a}}_{t}=a^{\prime }\right]-E_{t}\left[\bar{f}\left(a,c_{t}\right)\right]\right)}^{2}={∥M∥}_{F}^{2}, \end{array}$$

where the inequality follows by the application of Jensen’s inequality. We thus obtain that

$$\Gamma_{t}\leq \frac{\mathrm{Tr}{\left(M\right)}^{2}}{{∥M∥}_{F}^{2}}\leq m,$$

where the last inequality follows from Prop. 5 in [25]. From Lemma 3 in [19], we also obtain $\Gamma_{t}\leq 2\log\left(1+K\right)$. This results in the upper bound $\Gamma_{t}\leq \min\left\{m,2\log\left(1+K\right)\right\}$.

Using this and the bound of (A31) in (A28), we obtain

(A36) $$\begin{array}{c} R_{d,\mathrm{CB}}^{T}\leq \sqrt{2Tm\sigma^{2}\min\left\{m,2\left(1+\log K\right)\right\}\log\left(1+\frac{T\lambda }{m\sigma^{2}}\right)}. \end{array}$$

### Appendix B.4. Proof of Lemma 4

We now prove an upper bound on the term $R_{d,\mathrm{EE}1}^{T}$. To this end, let us define the following event:

(A37) $$\begin{array}{c} E:=\left\{∥\theta^{*}{∥}_{2}\leq \sqrt{2\lambda m\log\left(\frac{2m}{\delta }\right)}:=U\right\}. \end{array}$$

Note, that since $\theta^{*}∼N\left(\theta^{*}|0,\lambda I\right)$, we obtain that with probability at least 1−δ, the following inequality holds $∥\theta^{*}{∥}_{\infty }\leq \sqrt{2\lambda \log\left(\frac{2m}{\delta }\right)}$. Since $∥\theta^{*}{∥}_{2}\leq \sqrt{m}{∥\theta^{*}∥}_{\infty }$, the above inequality in turn implies the event E such that P(E)≥1−δ.

$$\begin{array}{cc} R_{d,\mathrm{EE}1}^{T} & \overset{\left(a\right)}{\leq }\sum_{t=1}^{T}E\left[\psi {\left(a_{t}^{*},{\hat{c}}_{t}|\gamma^{*}\right)}^{⊤}\theta^{*}-\psi {\left(a_{t}^{*},{\hat{c}}_{t}|H_{c,\hat{c}}\right)}^{⊤}\theta^{*}\right]\\ \; & =\sum_{t=1}^{T}E[\underset{:=\Delta \left(P\left(c_{t}|{\hat{c}}_{t},\gamma^{*}\right),P\left(c_{t}|{\hat{c}}_{t},H_{c,\hat{c}}\right)\right)}{\underset{︸}{E_{P(c_{t}|{\hat{c}}_{t},\gamma^{*})}\left[\phi {\left(a_{t}^{*},c_{t}\right)}^{⊤}\theta^{*}\right]-E_{P(c_{t}|{\hat{c}}_{t},H_{c,\hat{c}})}\left[\phi {\left(a_{t}^{*},c_{t}\right)}^{⊤}\theta^{*}\right]}}], \end{array}$$

(A38) $$\begin{array}{cc} \; & =\sum_{t=1}^{T}E[\Delta \left(P\left(c_{t}|{\hat{c}}_{t},\gamma^{*}\right),P\left(c_{t}|{\hat{c}}_{t},H_{c,\hat{c}}\right)\right)1\left\{E\right\}]+\sum_{t=1}^{T}E[\Delta \left(P\left(c_{t}|{\hat{c}}_{t},\gamma^{*}\right),P\left(c_{t}|{\hat{c}}_{t},H_{c,\hat{c}}\right)\right)1\left\{E^{c}\right\}] \end{array}$$

(A39) $$\begin{array}{cc} \; & \overset{\left(b\right)}{\leq }\sum_{t=1}^{T}E[\Delta \left(P\left(c_{t}|{\hat{c}}_{t},\gamma^{*}\right),P\left(c_{t}|{\hat{c}}_{t},H_{c,\hat{c}}\right)\right)1\left\{E\right\}]+2\delta TE[∥{\theta^{*}∥}_{2}\left|E^{c}\right], \end{array}$$

where the inequality (a) follows from the definition of ${\hat{a}}_{t}=\arg\max_{a\in A}\psi {\left(a,\hat{c}|H_{c,\hat{c}}\right)}^{⊤}\theta^{*}$, and 1{•} denotes the indicator function which takes value 1 when • is true and takes value 0 otherwise. The inequality in (b) follows by noting that

$$\begin{array}{cc} \; & E[\Delta \left(P\left(c_{t}|{\hat{c}}_{t},\gamma^{*}\right),P\left(c_{t}|{\hat{c}}_{t},H_{c,\hat{c}}\right)\right)I\left\{E^{c}\right\}]\\ & \leq E\left[{\left(E_{P(c_{t}|{\hat{c}}_{t},\gamma^{*})}\left[\phi \left(a_{t}^{*},c_{t}\right)\right]-E_{P(c_{t}|{\hat{c}}_{t},H_{c,\hat{c}})}\left[\phi \left(a_{t}^{*},c_{t}\right)\right]\right)}^{⊤}\theta^{*}1\left\{E^{c}\right\}\right]\\ \; & \leq E\left[∥E_{P(c_{t}|{\hat{c}}_{t},\gamma^{*})}\left[\phi \left(a_{t}^{*},c_{t}\right)\right]-E_{P(c_{t}|{\hat{c}}_{t},H_{c,\hat{c}})}\left[\phi \left(a_{t}^{*},c_{t}\right)\right]{∥}_{2}{∥\theta^{*}∥}_{2}1\left\{E^{c}\right\}\right]\\ \; & \leq 2E\left[∥\theta^{*}{∥}_{2}1\left\{E^{c}\right\}\right]=2P\left(E^{c}\right)E[∥\theta^{*}{∥}_{2}|E^{c}]\leq 2\delta E[∥\theta^{*}{∥}_{2}\left|E^{c}\right], \end{array}$$

where the last inequality is due to $P\left(E^{c}\right)=1-P\left(E\right)\leq \delta$. To obtain an upper bound on $E[∥\theta^{*}{∥}_{2}|E^{c}]$, we note that the following set of inequalities hold:

(A40) $$\begin{array}{cc} E[∥\theta^{*}{∥}_{2}|E^{c}] & \overset{\left(a\right)}{\leq }\sqrt{m}E[∥\theta^{*}{∥}_{\infty }|E^{c}]\overset{\left(b\right)}{=}\sqrt{m}E[∥\theta^{*}{∥}_{\infty }|∥\theta^{*}{∥}_{\infty }>u]\\ \; & =\sqrt{m}\sum_{i=1}^{m}P(∥\theta^{*}{∥}_{\infty }=|\theta_{i}^{*}|)E[∥\theta^{*}{∥}_{\infty }\left|∥\theta^{*}{∥}_{\infty }=|\theta_{i}^{*}|,∥\theta^{*}{∥}_{\infty }>u\right]\\ \; & \leq \sqrt{m}\sum_{i=1}^{m}E[\left|\theta_{i}^{*}\right|\left||\theta_{i}^{*}|>u\right]\overset{\left(c\right)}{=}2\sqrt{m}\sum_{i=1}^{m}\int_{x>u}xg\left(x\right)dx\\ \; & \overset{\left(d\right)}{=}-2\lambda \sqrt{m}\sum_{i=1}^{m}\int_{x>u}g^{\prime }\left(x\right)dx=2\lambda m^{3/2}g\left(u\right)=2\lambda m^{3/2}\frac{1}{\sqrt{2\pi \lambda }}\exp\left(-u^{2}/2\lambda \right)\\ \; & =\delta \sqrt{\frac{m\lambda }{2\pi }}, \end{array}$$

where (a) follows since ${∥\theta ∥}_{2}\leq \sqrt{m}{∥\theta ∥}_{\infty }$, (b) follows since $∥\theta^{*}{∥}_{2}>\sqrt{m}\sqrt{2\lambda \log\left(\frac{2m}{\delta }\right)}$ implies that $∥\theta^{*}{∥}_{\infty }>\sqrt{2\lambda \log\left(\frac{2m}{\delta }\right)}:=u$. The equality in (c) follows by noting that $|\theta_{i}^{*}|$, where $\theta_{i}^{*}∼N\left(0,\lambda \right)$, follows a folded Gaussian distribution with density $2g(\theta_{i}^{*})$ where $g\left(\theta_{i}^{*}\right)=\frac{1}{\sqrt{2\pi \lambda }}\exp\left(-\theta_{i}^{*2}/\left(2\lambda \right)\right)$ is the Gaussian density. The equality in (d) follows by noting that $xg\left(x\right)=-\lambda g^{\prime }\left(x\right)$, where $g^{\prime }\left(x\right)$ is the derivative of the Gaussian density. Thus, we have the following upper bound

(A41) $$\begin{array}{cc} \sum_{t=1}^{T}E\left[\Delta \left(P\left(c_{t}|{\hat{c}}_{t},\gamma^{*}\right),P\left(c_{t}|{\hat{c}}_{t},H_{c,\hat{c}}\right)\right)1\left\{E^{c}\right\}\right] & \leq 2T\delta^{2}\sqrt{\frac{m\lambda }{2\pi }}. \end{array}$$

We now obtain an upper bound on $\sum_{t=1}^{T}E\left[\Delta \left(P\left(c_{t}|{\hat{c}}_{t},\gamma^{*}\right),P\left(c_{t}|{\hat{c}}_{t},H_{c,\hat{c}}\right)\right)1\left\{E\right\}\right]$. To this end, note that

(A42) $$\begin{array}{cc} E\left[\Delta \left(P\left(c_{t}|{\hat{c}}_{t},\gamma^{*}\right),P\left(c_{t}|{\hat{c}}_{t},H_{c,\hat{c}}\right)\right)1\left\{E\right\}\right] & \leq P\left(E\right)E\left[\Delta \left(P\left(c_{t}|{\hat{c}}_{t},\gamma^{*}\right),P\left(c_{t}|{\hat{c}}_{t},H_{c,\hat{c}}\right)\right)|E\right]\\ \; & \leq E[\Delta \left(P\left(c_{t}|{\hat{c}}_{t},\gamma^{*}\right),P\left(c_{t}|{\hat{c}}_{t},H_{c,\hat{c}}\right)\right)\left|E\right]. \end{array}$$

Note, that under the event E, we have the following relation,

$$|\phi {\left(a_{t}^{*},c_{t}\right)}^{⊤}\theta^{*})|\leq ∥\phi \left(a_{t}^{*},c_{t}\right){∥}_{2}{∥\theta^{*}∥}_{2}\leq U,$$

whereby $\phi {\left(a_{t}^{*},c_{t}\right)}^{⊤}\theta^{*}$ is $U^{2}$-sub-Gaussian.

Consequently, applying Lemma A1 gives the following upper bound

$$\begin{array}{cc} \sum_{t=1}^{T}E[\Delta \left(P\left(c_{t}|{\hat{c}}_{t},\gamma^{*}\right),P\left(c_{t}|{\hat{c}}_{t},H_{c,\hat{c}}\right)\right)1\left\{E\right\}] & \leq \sum_{t=1}^{T}E\left[\sqrt{2U^{2}D_{\mathrm{KL}}(P\left(c_{t}|{\hat{c}}_{t},\gamma^{*}\right)∥P\left(c_{t}|{\hat{c}}_{t},H_{c,\hat{c}}\right)}\right]\\ \; & \leq \sqrt{2TU^{2}\sum_{t=1}^{T}E\left[D_{\mathrm{KL}}(P\left(c_{t}|{\hat{c}}_{t},\gamma^{*}\right)∥P\left(c_{t}|{\hat{c}}_{t},H_{c,\hat{c}}\right)\right]}\\ \; & \overset{\left(a\right)}{=}\sqrt{2TU^{2}\sum_{t=1}^{T}I\left(c_{t};\gamma^{*}|{\hat{c}}_{t},H_{c,\hat{c}}\right)} \end{array}$$

(A43) $$\begin{array}{cc} \; & \overset{\left(b\right)}{\leq }\sqrt{2TU^{2}\sum_{t=1}^{T}I\left(c_{t},{\hat{c}}_{t};\gamma^{*}|H_{c,\hat{c}}\right)} \end{array}$$

(A44) $$\begin{array}{cc} \; & \overset{\left(c\right)}{=}\sqrt{2TU^{2}I\left(H_{T,c,\hat{c}};\gamma^{*}\right)} \end{array}$$

where the equality in (a) follows by the definition of condition mutual information

$$I\left(c_{t};\gamma^{*}|{\hat{c}}_{t},H_{c,\hat{c}}\right):=E\left[D_{\mathrm{KL}}\left(P\left(c_{t},\gamma^{*}|{\hat{c}}_{t},H_{c,\hat{c}}\right)∥P\left(c_{t}|{\hat{c}}_{t},H_{c,\hat{c}}\right)P\left(\gamma^{*}|{\hat{c}}_{t},H_{c,\hat{c}}\right)\right)\right],$$

and inequality in (b) follows since $I\left(c_{t},{\hat{c}}_{t};\gamma^{*}|H_{c,\hat{c}}\right)=I\left({\hat{c}}_{t};\gamma^{*}|H_{c,\hat{c}}\right)+I\left(c_{t};\gamma^{*}|{\hat{c}}_{t},H_{c,\hat{c}}\right)\geq I\left(c_{t};\gamma^{*}|{\hat{c}}_{t},H_{c,\hat{c}}\right)$ due to the non-negativity of mutual information, and finally, the equality in (c) follows from the chain rule of mutual information.

We now analyze the mutual information $I(\gamma^{*};H_{T,c,\hat{c}})$ which can be written as

$$\begin{array}{cc} I(\gamma^{*};H_{T,c,\hat{c}}) & =H\left(\gamma^{*}\right)-H\left(\gamma^{*}|H_{T,c,\hat{c}}\right) \end{array}$$

(A45) $$\begin{array}{cc} \; & =\frac{1}{2}\log\left({\left(2\pi e\right)}^{d}\det\left(\Sigma_{\gamma }\right)\right)-\frac{1}{2}\log\left({\left(2\pi e\right)}^{d}\det\left(W^{-1}\right)\right) \end{array}$$

(A46) $$\begin{array}{cc} \; & =\frac{1}{2}\log\frac{\det(\Sigma_{\gamma })}{\det(W^{-1})} \end{array}$$

where $W=\left(T-1\right)\Sigma_{n}^{-1}+\Sigma_{\gamma }^{-1}=\Sigma_{\gamma }^{-1}\left(I+\left(T-1\right)\Sigma_{\gamma }\Sigma_{n}^{-1}\right)$. Using this, we can equivalently write

(A47) $$\begin{array}{cc} I(\gamma^{*};H_{T,c,\hat{c}}) & =\frac{1}{2}\log\frac{1}{\det({\left(I+\left(T-1\right)\Sigma_{\gamma }\Sigma_{n}^{-1}\right)}^{-1})} \end{array}$$

(A48) $$\begin{array}{cc} \; & =\frac{1}{2}\log\det\left(I+\left(T-1\right)\Sigma_{\gamma }\Sigma_{n}^{-1}\right). \end{array}$$

Under the assumption that $\Sigma_{\gamma }\Sigma_{n}^{-1}≻0$, we have $I+\left(T-1\right)\Sigma_{\gamma }\Sigma_{n}^{-1}≻0$, whereby using the determinant-trace inequality we obtain,

$$\begin{array}{cc} I(\gamma^{*};H_{T,c,\hat{c}}) & =\frac{1}{2}\log\left(\det(I+\left(T-1\right)\Sigma_{\gamma }\Sigma_{n}^{-1})\right)\\ & \leq \frac{d}{2}\log\left(\mathrm{Tr}(I+\left(T-1\right)\Sigma_{\gamma }\Sigma_{n}^{-1})/d)\right)\\ \; & =\frac{d}{2}\log\left(1+\left(T-1\right)\mathrm{Tr}\left(\Sigma_{\gamma }\Sigma_{n}^{-1}\right)/d\right)\\ \; & \leq \frac{d}{2}\log\left(1+T\mathrm{Tr}(\Sigma_{\gamma }\Sigma_{n}^{-1})/d\right). \end{array}$$

Using this in (A44), we obtain that

(A49) $$\begin{array}{c} \sum_{t=1}^{T}E[\Delta \left(P\left(c_{t}|{\hat{c}}_{t},\gamma^{*}\right),P\left(c_{t}|{\hat{c}}_{t},H_{c,\hat{c}}\right)\right)I\left\{E\right\}]\leq \sqrt{Td\log\left(1+T\mathrm{Tr}(\Sigma_{\gamma }\Sigma_{n}^{-1})/d\right)U^{2}}. \end{array}$$

Finally, using this in (A39), gives the following upper bound

(A50) $$\begin{array}{c} R_{d,\mathrm{EE}1}^{T}\leq \sqrt{2\lambda mTd\log\left(1+T\mathrm{Tr}(\Sigma_{\gamma }\Sigma_{n}^{-1})/d\right)\log\left(\frac{2m}{\delta }\right)}+2T\delta^{2}\sqrt{\frac{m\lambda }{2\pi }}. \end{array}$$

We finally note that same upper bound holds for the term $R_{d,\mathrm{EE}2}^{T}$.

## Appendix C. Linear-Gaussian Noisy Contextual Bandits with Unobserved True Contexts

### Appendix C.1. Derivation of Posterior Predictive Distribution

In this section, we derive the posterior predictive distribution $P(c_{t}|{\hat{c}}_{t},H_{t-1,\hat{c}})$ for Gaussian bandits with Gaussian context noise. To this end, we first derive the posterior $P(\gamma^{*}|H_{t-1,\hat{c}})$.

#### Appendix C.1.1. Derivation of Posterior $P(\gamma^{*}|H_{t-1,\hat{c}})$

Using Baye’s theorem, we have

$$\begin{array}{c} P\left(\gamma^{*}|H_{t-1,\hat{c}}\right)\propto P\left(\gamma^{*}\right)\prod_{\tau =1}^{t-1}P\left({\hat{c}}_{\tau }|\gamma^{*}\right), \end{array}$$

where $P({\hat{c}}_{\tau }|\gamma^{*})$ is derived in (A18). Subsequently, we have that

$$\begin{array}{cc} \log p(\gamma^{*}|H_{t-1},\hat{c}) & \propto -\frac{1}{2}\sum_{\tau =1}^{t-1}\left({\left({\hat{c}}_{\tau }-F\right)}^{⊤}G\left({\hat{c}}_{\tau }-F\right)\right)-\frac{1}{2}\left(\gamma^{*⊤}\Sigma_{\gamma }^{-1}\gamma^{*}\right)\\ \; & \propto -\frac{1}{2}(\gamma^{*⊤}\left(\left(t-1\right)G+\Sigma_{\gamma }^{-1}\right)\gamma^{*}-\gamma^{*⊤}\left(G{\hat{c}}_{1:t-1}-\left(t-1\right)\Sigma_{n}^{-1}{\left(M^{-1}\right)}^{⊤}\Sigma_{c}^{-1}\mu_{c}\right)\\ & -\left({\hat{c}}_{1:t-1}^{⊤}G-\left(t-1\right)\mu_{c}^{⊤}\Sigma_{c}^{-1}M^{-1}\Sigma_{n}^{-1}\right)\gamma^{*}), \end{array}$$

where we have denoted $\sum_{\tau =1}^{t-1}{\hat{c}}_{\tau }={\hat{c}}_{1:t-1}$. We then obtain

(A51) $$\begin{array}{cc} P(\gamma^{*}|H_{t-1,\hat{c}}) & =N({\tilde{M}}_{t},N_{t}^{-1})\;\mathrm{where}, \end{array}$$

(A52) $$\begin{array}{cc} N_{t} & =\left(t-1\right)G+\Sigma_{\gamma }^{-1} \end{array}$$

(A53) $$\begin{array}{cc} {\tilde{M}}_{t} & ={\left(N_{t}^{-1}\right)}^{⊤}\left(G{\hat{c}}_{1:t-1}-\left(t-1\right)\Sigma_{n}^{-1}{\left(M^{-1}\right)}^{⊤}\Sigma_{c}^{-1}\mu_{c}\right). \end{array}$$

#### Appendix C.1.2. Derivation of $P(c_{t}|{\hat{c}}_{t},H_{t-1,\hat{c}})$

The derivation of posterior predictive distribution $P(c_{t}|{\hat{c}}_{t},H_{t-1,\hat{c}})$ follows in a similar line as that in Appendix B.2.4. We start the derivation by noting that $P\left(c_{t}|{\hat{c}}_{t},H_{t-1,\hat{c}}\right)=E_{P(\gamma^{*}|H_{t-1,\hat{c}})}\left[P\left(c_{t}|{\hat{c}}_{t},\gamma^{*}\right)\right]$.

We have

(A54) $$\begin{array}{cc} \; & \log(P\left(\gamma^{*}|H_{t-1,\hat{c}}\right)P\left(c_{t}|{\hat{c}}_{t},\gamma^{*}\right))\\ & \propto -\frac{1}{2}\left({\left(c_{t}-A_{t}\right)}^{⊤}M\left(c_{t}-A_{t}\right)+{\left(\gamma^{*}-{\tilde{M}}_{t}\right)}^{⊤}N\left(\gamma^{*}-{\tilde{M}}_{t}\right)\right)\\ \; & =-\frac{1}{2}({\left(M^{-1}{)}^{⊤}\Sigma_{n}^{-1}\gamma^{*}+c_{t}-D-E_{t}\right)}^{⊤}M\left({\left(M^{-1}\right)}^{⊤}\Sigma_{n}^{-1}\gamma^{*}+c_{t}-D-E_{t}\right)\\ & +{\left(\gamma^{*}-{\tilde{M}}_{t}\right)}^{⊤}N_{t}\left(\gamma^{*}-{\tilde{M}}_{t}\right))\\ \; & =-\frac{1}{2}\left({\left(\gamma^{*}-J_{t}\right)}^{⊤}H_{t}\left(\gamma^{*}-J_{t}\right)-J_{t}^{⊤}H_{t}J_{t}+{\left(c_{t}-D-E_{t}\right)}^{⊤}M\left(c_{t}-D-E_{t}\right)+{\tilde{M}}_{t}^{⊤}N_{t}{\tilde{M}}_{t}\right), \end{array}$$

where $A_{t}$ is defined in (A14), *M* is defined in (A13), ${\tilde{M}}_{t}$ in (A53), *N* in (A52), $D={\left(M^{-1}\right)}^{⊤}\Sigma_{c}^{-1}\mu_{c}$, $E_{t}={\left(M^{-1}\right)}^{⊤}\Sigma_{n}^{-1}{\hat{c}}_{t}$, $H_{t}=\Sigma_{n}^{-1}{\left(M^{-1}\right)}^{⊤}\Sigma_{n}^{-1}+N_{t}$ and $J_{t}={\left(H_{t}^{-1}\right)}^{⊤}\left(\Sigma_{n}^{-1}\left(-c_{t}+D+E_{t}\right)+N_{t}{\tilde{M}}_{t}\right)$.

Subsequently, we have

(A55) $$\begin{array}{cc} \log p(c_{t}|{\hat{c}}_{t},H_{t-1,\hat{c}}) & \propto -\frac{1}{2}\left(-J_{t}^{⊤}H_{t}J_{t}+{\left(c_{t}-D-E_{t}\right)}^{⊤}M\left(c_{t}-D-E_{t}\right)\right)\\ \; & \propto -\frac{1}{2}(c_{t}^{⊤}\left(M-\Sigma_{n}^{-1}{\left(H_{t}^{-1}\right)}^{⊤}\Sigma_{n}^{-1}\right)c_{t}-c_{t}^{⊤}\left(M\left(D+E_{t}\right)-\Sigma_{n}^{-1}{\left(H_{t}^{-1}\right)}^{⊤}L_{t}^{⊤}\right) \end{array}$$

(A56) $$\begin{array}{cc} & -\left({\left(D+E_{t}\right)}^{⊤}M-L_{t}\left(H_{t}^{-1}\right)\Sigma_{n}^{-1}\right), \end{array}$$

where $L_{t}={\left(D+E_{t}\right)}^{⊤}\Sigma_{n}^{-1}+{\tilde{M}}_{t}^{⊤}N_{t}$. Thus, we have,

(A57) $$\begin{array}{cc} P(c_{t}|{\hat{c}}_{t},H_{t-1,\hat{c}}) & =N(c_{t}|V_{t},R_{t}^{-1}),\;\mathrm{where} \end{array}$$

(A58) $$\begin{array}{cc} \; & R_{t}=M-\Sigma_{n}^{-1}{\left(H_{t}^{-1}\right)}^{⊤}\Sigma_{n}^{-1} \end{array}$$

(A59) $$\begin{array}{cc} \; & V_{t}={\left(R_{t}^{-1}\right)}^{⊤}\left(M\left(D+E_{t}\right)-\Sigma_{n}^{-1}{\left(H_{t}^{-1}\right)}^{⊤}L_{t}^{⊤}\right). \end{array}$$

#### Appendix C.1.3. Evaluating the KL Divergence between the True Posterior $P_{t}\left(\theta^{*}\right)$ and Sampling Distribution ${\bar{P}}_{t}\left(\theta^{*}\right)$

In this subsection, we analyze the true posterior distribution $P_{t}\left(\theta^{*}\right):=P\left(\theta^{*}|H_{t-1,r,a,\hat{c}}\right)$ and the approximate sampling distribution ${\bar{P}}_{t}\left(\theta^{*}\right):=\bar{P}\left(\theta^{*}|H_{t-1,r,a,\hat{c}}\right)$, and derive the KL divergence $D_{\mathrm{KL}}\left(P_{t}\left(\theta^{*}\right)∥{\bar{P}}_{t}\left(\theta^{*}\right)\right)$ between them. To see this, note that from Bayes’s theorem, we have the following joint probability distribution

(A60) $$\begin{array}{cc} P(\theta^{*},H_{t-1,r,\hat{c}}|H_{t-1,a}) & =P\left(\theta^{*}\right)\underset{:=P(H_{t-1,r,\hat{c}}|H_{t-1,a},\theta^{*})}{\underset{︸}{E_{P(\gamma^{*})}\left[\prod_{\tau =1}^{t-1}E_{P(c_{\tau })}\left[P\left({\hat{c}}_{\tau }|c_{\tau },\gamma^{*}\right)P\left(r_{\tau }|a_{\tau },c_{\tau },\theta^{*}\right)\right]\right]}} \end{array}$$

(A61) $$\begin{array}{cc} \; & =P\left(\theta^{*}\right)P\left(H_{t-1,r,\hat{c}}|H_{t-1,a},\theta^{*}\right) \end{array}$$

whereby we obtain that

(A62) $$\begin{array}{c} P_{t}\left(\theta^{*}\right)\propto P\left(\theta^{*}\right)P\left(H_{t-1,r,\hat{c}}|H_{t-1,a},\theta^{*}\right). \end{array}$$

In particular, for general feature maps ϕ(a,c), the distribution $P(H_{t-1,r,\hat{c}}|H_{t-1,a},\theta^{*})$ cannot be exactly evaluated, even under Gaussian assumptions, resulting in the posterior $P_{t}\left(\theta^{*}\right)$ to be intractable, in general.

In contrast to this, our approximate TS-algorithm scheme assumes the following joint probability distribution,

(A63) $$\begin{array}{cc} \bar{P}\left(\theta^{*},H_{t-1,r,\hat{c}}|H_{t-1,a}\right) & =P\left(\theta^{*}\right)\underset{:=\bar{P}\left(H_{t-1,r,\hat{c}}|H_{t-1,a},\theta^{*}\right)}{\underset{︸}{E_{P(\gamma^{*})}\left[\prod_{\tau =1}^{t-1}E_{P(c_{\tau })}\left[P\left({\hat{c}}_{\tau }|c_{\tau },\gamma^{*}\right)\bar{P}\left(r_{\tau }|a_{\tau },{\hat{c}}_{\tau },H_{\tau -1,\hat{c}},\theta^{*}\right)\right]\right]}} \end{array}$$

(A64) $$\begin{array}{cc} \; & =P\left(\theta^{*}\right)\bar{P}\left(H_{t-1,r,\hat{c}}|H_{t-1,a},\theta^{*}\right), \end{array}$$

where

$$\bar{P}\left(r_{\tau }|a_{\tau },{\hat{c}}_{\tau },H_{\tau -1,\hat{c}},\theta^{*}\right)=N\left(\psi \left(a_{\tau },{\hat{c}}_{\tau }|H_{\tau -1,\hat{c}}\right),\sigma^{2}\right).$$

Consequently, we have

(A65) $$\begin{array}{cc} {\bar{P}}_{t}\left(\theta^{*}\right) & \propto P\left(\theta^{*}\right)\bar{P}\left(H_{t-1,r,\hat{c}}|H_{t-1,a},\theta^{*}\right)\\ \; & =P\left(\theta^{*}\right)\left(\prod_{\tau =1}^{t-1}\bar{P}\left(r_{\tau }|a_{\tau },{\hat{c}}_{\tau },H_{\tau -1,\hat{c}},\theta^{*}\right)\right)\left(E_{P(\gamma^{*})}\left[\prod_{\tau =1}^{t-1}[P\left({\hat{c}}_{\tau }|\gamma^{*}\right)\right]\right). \end{array}$$

As a result, we can upper bound the KL divergence $D_{\mathrm{KL}}\left(P_{t}\left(\theta^{*}\right)∥{\bar{P}}_{t}\left(\theta^{*}\right)\right)$ as

$$\begin{array}{c} D_{\mathrm{KL}}\left(P_{t}\left(\theta^{*}\right)∥{\bar{P}}_{t}\left(\theta^{*}\right)\right)\\ \leq D_{\mathrm{KL}}\left(P\left(\theta^{*},H_{t-1,r,\hat{c}}|H_{t-1,a}\right)∥\bar{P}\left(\theta^{*},H_{t-1,r,\hat{c}}|H_{t-1,a}\right)\right)\\ =D_{\mathrm{KL}}\left(P\left(\theta^{*}\right)P\left(H_{t-1,r,\hat{c}}|\theta^{*},H_{t-1,a}\right)∥P\left(\theta^{*}\right)\bar{P}\left(H_{t-1,r,\hat{c}}|H_{t-1,a},\theta^{*}\right)\right)\\ =E_{P(\theta^{*})}\left[D_{\mathrm{KL}}\left(P\left(H_{t-1,r,\hat{c}}|\theta^{*},H_{t-1,a}\right)∥\bar{P}\left(H_{t-1,r,\hat{c}}|H_{t-1,a},\theta^{*}\right)\right)\right]\\ \overset{\left(a\right)}{\leq }E_{P\left(\theta^{*}\right)P\left(\gamma^{*}\right)}E_{P(H_{t-1,c})}\left[D_{\mathrm{KL}}\left(\prod_{\tau =1}^{t-1}P\left({\hat{c}}_{\tau }|c_{\tau },\gamma^{*}\right)P\left(r_{\tau }|a_{\tau },c_{\tau },\theta^{*}\right)∥\prod_{\tau =1}^{t-1}P\left({\hat{c}}_{\tau }|c_{\tau },\gamma^{*}\right)\bar{P}\left(r_{\tau }|a_{\tau },H_{\tau ,\hat{c}},\theta^{*}\right)\right)\right]\\ =E_{P(\theta^{*})}E_{P(\gamma^{*})}E_{P(H_{t-1,c})}\left[\sum_{\tau =1}^{t-1}E_{P(H_{\tau -1,\hat{c}}|H_{\tau -1,c},\gamma^{*})}\left[D_{\mathrm{KL}}\left(P\left(r_{\tau }|a_{\tau },c_{\tau },\theta^{*}\right)∥\bar{P}\left(r_{\tau }|a_{\tau },{\hat{c}}_{\tau },H_{\tau -1,\hat{c}},\theta^{*}\right)\right)\right]\right] \end{array}$$

(A66) $$\begin{array}{cc} \; & \overset{\left(b\right)}{=}E_{P(\theta^{*})}E_{P(\gamma^{*})}E_{P(H_{t-1,c})}\left[\sum_{\tau =1}^{t-1}E_{P(H_{\tau -1,\hat{c}}|H_{\tau -1,c},\gamma^{*})}\left[\frac{{\left(\phi {\left(a_{\tau },c_{\tau }\right)}^{⊤}\theta^{*}-\psi {\left({\hat{c}}_{\tau },a_{\tau }|H_{\tau -1,\hat{c}}\right)}^{⊤}\theta^{*}\right)}^{2}}{2\sigma^{2}}\right]\right],\\ \; & =E_{P(\theta^{*})}E_{P(\gamma^{*})}E_{P(H_{t-1,c})}\left[\sum_{\tau =1}^{t-1}E_{P(H_{\tau -1,\hat{c}}|H_{\tau -1,c},\gamma^{*})}\left[\frac{|{\left(\phi \left(a_{\tau },c_{\tau }\right)-\psi \left({\hat{c}}_{\tau },a_{\tau }|H_{\tau -1,\hat{c}}\right)\right)}^{⊤}\theta^{*}{|}^{2}}{2\sigma^{2}}\right]\right],\\ \; & \overset{\left(c\right)}{\leq }E_{P(\theta^{*})}E_{P(\gamma^{*})}E_{P(H_{t-1,c})}\left[\sum_{\tau =1}^{t-1}E_{P(H_{\tau -1,\hat{c}}|H_{\tau -1,c},\gamma^{*})}\left[\frac{∥(\phi \left(a_{\tau },c_{\tau }\right)-\psi \left({\hat{c}}_{\tau },a_{\tau }|H_{\tau -1,\hat{c}}\right){∥}_{2}^{2}∥\theta^{*}{∥}_{2}^{2}}{2\sigma^{2}}\right]\right]\\ \; & \overset{\left(d\right)}{\leq }E_{P(\theta^{*})}E_{P(\gamma^{*})}E_{P(H_{t-1,c})}\left[\sum_{\tau =1}^{t-1}E_{P(H_{\tau -1,\hat{c}}|H_{\tau -1,c},\gamma^{*})}\left[\frac{4∥\theta^{*}{∥}_{2}^{2}}{2\sigma^{2}}\right]\right]\\ \; & =\sum_{\tau =1}^{t-1}E_{P(\theta^{*})}\left[\frac{2∥\theta^{*}{∥}_{2}^{2}}{\sigma^{2}}\right]\overset{\left(e\right)}{=}\frac{2(t-1)\lambda m}{\sigma^{2}}. \end{array}$$

In the above series of relationships,

- equality in (a) follows by noting that
  $$\bar{P}\left(H_{t-1,r,\hat{c}}|H_{t-1,a},\theta^{*}\right)=E_{P(\gamma^{*})}E_{P(H_{t-1,c})}\left[\prod_{\tau =1}^{t-1}P\left({\hat{c}}_{\tau }|c_{\tau },\gamma^{*}\right)\bar{P}\left(r_{\tau }|a_{\tau },{\hat{c}}_{\tau },\theta^{*}\right)\right]$$
  and
  $$P\left(H_{t-1,r,\hat{c}}|H_{t-1,a},\theta^{*}\right)=E_{P(\gamma^{*})}E_{P(H_{t-1,c})}\left[\prod_{\tau =1}^{t-1}P\left({\hat{c}}_{\tau }|c_{\tau },\gamma^{*}\right)P\left(r_{\tau }|a_{\tau },c_{\tau },\theta^{*}\right)\right],$$
  and applying Jensen’s inequality on the jointly convex KL divergence,
- equality in (b) follows from evaluating the KL divergence between two Gaussian distributions with same variance $\sigma^{2}$ and with means $\phi {\left(a_{\tau },c_{\tau }\right)}^{⊤}\theta^{*}$ and $\psi {\left({\hat{c}}_{\tau },a_{\tau }|H_{\tau -1,\hat{c}}\right)}^{⊤}\theta^{*}$ respectively,
- inequality in (c) follows from application of Cauchy–Schwarz inequality,
- inequality in (d) follows from Assumption 1,
- inequality in (e) follows from
  $$E_{P(\theta^{*})}[∥\theta^{*}{∥}_{2}^{2}]=\sum_{j=1}^{m}E\left[{\left(\theta_{j}^{*}\right)}^{2}\right]=\lambda m$$
  since $P\left(\theta^{*}\right)=N\left(0,\lambda I\right)$.

### Appendix C.2. Proof of Lemma 1

For notational simplicity, throughout this section we use $\psi \left(a\right):=\psi \left(a,{\hat{c}}_{t}|H_{\hat{c}}\right)=E_{P(c_{t}|{\hat{c}}_{t},H_{t-1,\hat{c}})}\left[\phi \left(a,c_{t}\right)\right]$ to denote the expected feature map. Furthermore, we use $F_{t}=H_{t-1,r,a,\hat{c}}\cup {\hat{c}}_{t}$.

We start by distinguishing the true and approximated posterior distributions. Recall that $P_{t}\left(\theta^{*}\right):=P\left(\theta^{*}|H_{t-1,r,a,\hat{c}}\right)$ denotes the true posterior and ${\bar{P}}_{t}\left(\theta^{*}\right):=\bar{P}\left(\theta^{*}|H_{t-1,r,a,\hat{c}}\right)$ denotes the approximated posterior. We then denote $P_{t}\left({\hat{a}}_{t},\theta^{*}\right):=P\left({\hat{a}}_{t},\theta^{*}|F_{t}\right)$ as the distribution of ${\hat{a}}_{t}$ and $\theta^{*}$ conditioned on $F_{t}$, while ${\bar{P}}_{t}\left({\hat{a}}_{t},\theta^{*}\right):=\bar{P}\left({\hat{a}}_{t},\theta^{*}|F_{t}\right)$ denote the distribution of ${\hat{a}}_{t}$ and $\theta^{*}$ under the sampling distribution. Furthermore, we have that ${\bar{P}}_{t}\left(a_{t},\theta^{*}\right)={\bar{P}}_{t}\left(a_{t}\right){\bar{P}}_{t}\left(\theta^{*}\right)=P_{t}\left(a_{t}\right){\bar{P}}_{t}\left(\theta^{*}\right)$. We start by decomposing $R_{\mathrm{CB}}^{T}$ into the following three differences,

(A67) $$\begin{array}{cc} R_{\mathrm{CB}}^{T} & =\sum_{t=1}^{T}E\left[E_{P_{t}\left({\hat{a}}_{t},\theta^{*}\right)}\left[\psi {\left({\hat{a}}_{t}\right)}^{⊤}\theta^{*}\right]-E_{P_{t}\left(a_{t},\theta^{*}\right)}\left[\psi {\left(a_{t}\right)}^{⊤}\theta^{*}\right]\right]\\ \; & =\sum_{t=1}^{T}E[\underset{:={\mathrm{Term}}_{1}}{\underset{︸}{E_{{\bar{P}}_{t}\left({\hat{a}}_{t},\theta^{*}\right)}\left[\psi {\left({\hat{a}}_{t}\right)}^{⊤}\theta^{*}\right]-E_{{\bar{P}}_{t}\left(a_{t},\theta^{*}\right)}\left[\psi {\left(a_{t}\right)}^{⊤}\theta^{*}\right]}}+\underset{:={\mathrm{Term}}_{2}}{\underset{︸}{E_{P_{t}\left({\hat{a}}_{t},\theta^{*}\right)}\left[\psi {\left({\hat{a}}_{t}\right)}^{⊤}\theta^{*}\right]-E_{{\bar{P}}_{t}\left({\hat{a}}_{t},\theta^{*}\right)}\left[\psi {\left({\hat{a}}_{t}\right)}^{⊤}\theta^{*}\right]]}}\\ & +\underset{:={\mathrm{Term}}_{3}}{\underset{︸}{E_{{\bar{P}}_{t}\left(a_{t},\theta^{*}\right)}\left[\psi {\left(a_{t}\right)}^{⊤}\theta^{*}\right]-E_{P_{t}\left(a_{t},\theta^{*}\right)}\left[\psi {\left(a_{t}\right)}^{⊤}\theta^{*}\right]]}} \end{array}$$

We will separately upper bound each of the three terms in the above decomposition.

#### Appendix C.2.1. Upper Bound on ${\mathrm{Term}}_{2}$

To obtain an upper bound on ${\mathrm{Term}}_{2}$, note that the following equivalence holds $E_{P_{t}\left({\hat{a}}_{t},\theta^{*}\right)}\left[\psi {\left({\hat{a}}_{t}\right)}^{⊤}\theta^{*}\right]=E_{P_{t}\left(\theta^{*}\right)}\left[\max_{a}\psi {\left(a\right)}^{⊤}\theta^{*}\right]$. Using this, we can rewrite ${\mathrm{Term}}_{2}$ as

(A68) $$\begin{array}{c} {\mathrm{Term}}_{2}=E_{P_{t}\left(\theta^{*}\right)}\left[\max_{a}\psi {\left(a\right)}^{⊤}\theta^{*}\right]-E_{{\bar{P}}_{t}\left(\theta^{*}\right)}\left[\max_{a}\psi {\left(a\right)}^{⊤}\theta^{*}\right]. \end{array}$$

Note, here that when $\theta^{*}∼{\bar{P}}_{t}\left(\theta^{*}\right)$, for each a∈A, we have that $z_{a}=\psi {\left(a\right)}^{⊤}\theta^{*}$ follows Gaussian distribution $N(z_{a}|\psi {\left(a\right)}^{⊤}\mu_{t-1},\psi {\left(a\right)}^{⊤}\Sigma_{t-1}^{-1}\psi \left(a\right))$ with mean $\psi {\left(a\right)}^{⊤}\mu_{t-1}$ and variance $\psi {\left(a\right)}^{⊤}\Sigma_{t-1}^{-1}\psi \left(a\right)$, where $\mu_{t-1}$ and $\Sigma_{t-1}$ are as defined in (18) and (17), respectively. Thus, $E_{{\bar{P}}_{t}\left(\theta^{*}\right)}\left[\max_{a}z_{a}\right]$ is the average of maximum of Gaussian random variables. We can then apply Lemma A2 with $P\left(x\right)=P_{t}\left(\theta^{*}\right)$, $Q\left(x\right)={\bar{P}}_{t}\left(\theta^{*}\right)$, n=|A|=K, $\mu_{i}=\psi {\left(a\right)}^{⊤}\mu_{t-1}$ and $\sigma_{i}=\psi {\left(a\right)}^{⊤}\Sigma_{t-1}^{-1}\psi \left(a\right)$ to obtain that

(A69) $$\begin{array}{cc} {\mathrm{Term}}_{2} & \leq \sqrt{2(\log K+D_{\mathrm{KL}}\left(P_{t}\left(\theta^{*}\right)∥{\bar{P}}_{t}\left(\theta^{*}\right)\right)\max_{a}\psi {\left(a\right)}^{⊤}\Sigma_{t}^{-1}\psi \left(a\right)}. \end{array}$$

Using this, we obtain that

(A70) $$\begin{array}{cc} \sum_{t=1}^{T}E\left[{\mathrm{Term}}_{2}\right] & \leq E\left[\sum_{t=1}^{T}\sqrt{2\left(\log K+D_{\mathrm{KL}}\left(P_{t}\left(\theta^{*}\right)∥{\bar{P}}_{t}\left(\theta^{*}\right)\right)\right)\max_{a}\psi {\left(a\right)}^{⊤}\Sigma_{t}^{-1}\psi \left(a\right)}\right]\\ \; & \overset{\left(a\right)}{\leq }\sqrt{\left(\sum_{t=1}^{T}E\left[2(\log K+D_{\mathrm{KL}}\left(P_{t}\left(\theta^{*}\right)∥{\bar{P}}_{t}\left(\theta^{*}\right)\right))\right]\right)\left(\sum_{t=1}^{T}E\left[\max_{a}\psi {\left(a\right)}^{⊤}\Sigma_{t}^{-1}\psi \left(a\right)\right]\right)},\\ \; & \overset{\left(b\right)}{\leq }\sqrt{2\lambda T\left(\sum_{t=1}^{T}E\left[\log K+D_{\mathrm{KL}}\left(P_{t}\left(\theta^{*}\right)∥{\bar{P}}_{t}\left(\theta^{*}\right)\right)\right]\right)}\\ \; & \overset{\left(c\right)}{\leq }\sqrt{2\lambda T\left(T\log K+\sum_{t=1}^{T}2\left(t-1\right)\frac{\lambda m}{\sigma^{2}}\right)}\\ \; & =\sqrt{2\lambda T^{2}\log K+\frac{4\lambda^{2}T\left(T^{2}-T\right)m}{2\sigma^{2}}}\\ \; & \leq \sqrt{2\lambda T^{2}\log K+\frac{2\lambda^{2}T^{3}m}{\sigma^{2}}}, \end{array}$$

where the inequality in (a) follows from Cauchy–Schwarz inequality, and the inequality in (b) follows since

$$\begin{array}{c} \sum_{t=1}^{T}E\left[\max_{a}\psi {\left(a\right)}^{⊤}\Sigma_{t}^{-1}\psi \left(a\right)\right]\leq \sum_{t=1}^{T}E\left[\max_{a}\psi {\left(a\right)}^{⊤}\left(\lambda I\right)\psi \left(a\right)\right]\leq \lambda T, \end{array}$$

which follows since $\Sigma_{t}^{-1}\leq \lambda I$ and ∥ψ(a)∥≤1. The inequality in (c) follows from (A66).

If $\lambda \leq \frac{\sigma^{2}}{T}$, we obtain that

(A71) $$\begin{array}{c} \sum_{t=1}^{T}E\left[{\mathrm{Term}}_{2}\right]\leq \sqrt{2T\sigma^{2}\left(\log\left(K\right)+m\right)}. \end{array}$$

#### Appendix C.2.2. Upper Bound on ${\mathrm{Term}}_{3}$

We can bound ${\mathrm{Term}}_{3}$ by observing that

(A72) $$\begin{array}{cc} {\mathrm{Term}}_{3} & =E_{P_{t}\left(a_{t}\right)}{\left[\psi \left(a_{t}\right)\right]}^{⊤}\left(E_{{\bar{P}}_{t}\left(\theta^{*}\right)}\left[\theta^{*}\right]-E_{P_{t}\left(\theta^{*}\right)}\left[\theta^{*}\right]\right) \end{array}$$

(A73) $$\begin{array}{cc} \; & =E_{{\bar{P}}_{t}\left(\theta^{*}\right)}\left[\Psi_{t}^{⊤}\theta^{*}\right]-E_{P_{t}\left(\theta^{*}\right)}\left[\Psi_{t}^{⊤}\theta^{*}\right] \end{array}$$

where we used $\Psi_{t}=E_{P_{t}\left(a_{t}\right)}\left[\psi \left(a_{t}\right)\right]$. Note, that for $\theta^{*}∼{\bar{P}}_{t}\left(\theta^{*}\right)$, the random variable $\Psi_{t}^{⊤}\theta^{*}$ is Gaussian with mean $\Psi_{t}^{⊤}\mu_{t-1}$ and variance $\Psi_{t}^{⊤}\Sigma_{t-1}^{-1}\Psi_{t}$. Consequently, $\Psi_{t}^{⊤}\theta^{*}$ is also $\Psi_{t}^{⊤}\Sigma_{t-1}^{-1}\Psi_{t}$-sub-Gaussian according to Definition A.1. By using Lemma A1, we then obtain that

(A74) $$\begin{array}{c} |{\mathrm{Term}}_{3}|\leq \sqrt{2\left(\Psi_{t}^{⊤}\Sigma_{t}^{-1}\Psi_{t}\right)D_{\mathrm{KL}}\left(P_{t}\left(\theta^{*}\right)∥{\bar{P}}_{t}\left(\theta^{*}\right)\right)}. \end{array}$$

Using Cauchy–Schwarz inequality then yields that

$$\begin{array}{cc} E[\sum_{t=1}^{T}|{\mathrm{Term}}_{3}|] & \leq \sqrt{\left(\sum_{t=1}^{T}E\left[\Psi_{t}^{⊤}\overset{-1}{\underset{t}{\Sigma }}\Psi_{t}\right]\right)\left(\sum_{t=1}^{T}E\left[2D_{\mathrm{KL}}\left(P_{t}\left(\theta^{*}\right)∥{\bar{P}}_{t}\left(\theta^{*}\right)\right)\right]\right)}\\ \; & \leq \sqrt{2\lambda T\frac{\lambda T^{2}m}{\sigma^{2}}}=\sqrt{2\lambda^{2}T^{3}\frac{m}{\sigma^{2}}}. \end{array}$$

where the second inequality follows from (A66). As before, if $\lambda \leq \frac{\sigma^{2}}{T}$, we then obtain that

(A75) $$\begin{array}{cc} E[\sum_{t=1}^{T}|{\mathrm{Term}}_{3}|] & \leq \sqrt{2Tm\sigma^{2}}. \end{array}$$

#### Appendix C.2.3. Upper Bound on ${\mathrm{Term}}_{1}$

Note, that in ${\mathrm{Term}}_{1}$, ${\bar{P}}_{t}\left({\hat{a}}_{t}\right)={\bar{P}}_{t}\left(a_{t}\right)=P_{t}\left(a_{t}\right)$, whereby the posterior is matched. Hence, one can apply bounds from conventional contextual Thompson Sampling here. For simplicity, we denote ${\bar{E}}_{t}\left[\cdot \right]=E_{\bar{P}}\left[\cdot |F_{t}\right]$ to denote the expectation with respect to ${\bar{P}}_{t}\left(a,\theta \right)$. To this end, as in the proof of Lemma 3, we start by defining an information ratio,

(A76) $$\begin{array}{c} \Gamma_{t}=\frac{{{\mathrm{Term}}_{1}}^{2}}{{\bar{E}}_{t}\left[{\left(\psi {\left(a_{t}\right)}^{⊤}\theta^{*}-\psi {\left(a_{t}\right)}^{⊤}\mu_{t})\right)}^{2}\right]:=\Lambda_{t}}, \end{array}$$

using which we obtain the upper bound on ${\mathrm{Term}}_{1}$ as

(A77) $$\begin{array}{c} \sum_{t=1}^{T}E\left[{\mathrm{Term}}_{1}\right]\leq E\left[\sum_{t=1}^{T}\sqrt{\Gamma_{t}\Lambda_{t}}\right]\leq \sqrt{\left(\sum_{t=1}^{T}E\left[\Gamma_{t}\right]\right)\left(\sum_{t=1}^{T}E\left[\Lambda_{t}\right]\right)} \end{array}$$

by the Cauchy–Schwarz inequality.

Furthermore, we have

(A78) $$\begin{array}{cc} \Lambda_{t}={\bar{E}}_{t}\left[{\left(\psi {\left(a_{t}\right)}^{⊤}\left(\theta^{*}-\mu_{t}\right)\right)}^{2}\right] & ={\bar{E}}_{t}\left[\psi {\left(a_{t}\right)}^{⊤}\left(\theta^{*}-\mu_{t-1}\right){\left(\theta^{*}-\mu_{t-1}\right)}^{⊤}\psi \left(a_{t}\right)\right]\\ \; & ={\bar{E}}_{t}\left[\psi {\left(a_{t}\right)}^{⊤}\Sigma_{t-1}^{-1}\psi \left(a_{t}\right)\right]={\bar{E}}_{t}[∥\psi \left(a_{t}\right){∥}_{\Sigma_{t-1}^{-1}}], \end{array}$$

where $\mu_{t-1}$ and $\Sigma_{t-1}$ are defined as in (18) andd (17). Subsequently, using elliptical potential lemma, we obtain

(A79) $$\begin{array}{c} \sum_{t=1}^{T}\Lambda_{t}\leq 2m\sigma^{2}\log\left(1+\frac{(T)\lambda }{m\sigma^{2}}\right). \end{array}$$

To obtain an upper bound on the information ratio $\Gamma_{t}$, we define $\bar{f}\left(\theta^{*},a\right)=\psi {\left(a\right)}^{⊤}\theta^{*}$ and $\bar{f}\left(a\right)=\psi {\left(a\right)}^{⊤}\mu_{t-1}$ and let

(A80) $$\begin{array}{c} M_{a,a^{\prime }}=\sum_{a,a^{\prime }}\sqrt{{\bar{P}}_{t}\left(a_{t}=a\right){\bar{P}}_{t}\left({\hat{a}}_{t}=a^{\prime }\right)}\left({\bar{E}}_{t}\left[\bar{f}\left(\theta^{*},a\right)|{\hat{a}}_{t}=a^{\prime }\right]-\bar{f}\left(a\right)\right). \end{array}$$

It is easy to see that

(A81) $$\begin{array}{cc} {\mathrm{Term}}_{1} & ={\bar{E}}_{t}\left[\bar{f}\left({\hat{a}}_{t},\theta^{*}\right)\right]-{\bar{E}}_{t}\left[\bar{f}\left(a_{t}\right)\right]\\ \; & =\sum_{a^{\prime }}{\bar{P}}_{t}\left({\hat{a}}_{t}=a^{\prime }\right)\left({\bar{E}}_{t}\left[\bar{f}\left(\theta^{*},a^{\prime }\right)|{\hat{a}}_{t}=a^{\prime }\right]-{\bar{E}}_{t}\left[\bar{f}\left({\hat{a}}_{t}\right)\right]\right)\\ \; & =\sum_{a^{\prime }}{\bar{P}}_{t}\left({\hat{a}}_{t}=a^{\prime }\right)\left({\bar{E}}_{t}\left[\bar{f}\left(\theta^{*},a^{\prime }\right)|{\hat{a}}_{t}=a^{\prime }\right]-\bar{f}\left(a^{\prime }\right)\right)=\mathrm{Tr}\left(M\right), \end{array}$$

where the second and last equality follows since ${\bar{P}}_{t}\left(a_{t}\right)={\bar{P}}_{t}\left({\hat{a}}_{t}\right)$. Similarly, we can relate $\Lambda_{t}$ with the matrix $\left(M_{a,a^{\prime }}\right)$ as

(A82) $$\begin{array}{cc} \Lambda_{t} & ={\bar{E}}_{t}\left[{\left(\bar{f}\left(\theta^{*},a_{t}\right)-\bar{f}\left(a_{t}\right)\right)}^{2}\right]\\ \; & =\sum_{a}{\bar{P}}_{t}\left(a_{t}=a\right){\bar{E}}_{t}\left[{\left(\bar{f}\left(\theta^{*},a\right)-\bar{f}\left(a\right)\right)}^{2}\right]\\ \; & =\sum_{a,a^{\prime }}{\bar{P}}_{t}\left(a_{t}=a\right){\bar{P}}_{t}\left({\hat{a}}_{t}=a^{\prime }\right){\bar{E}}_{t}\left[{\left(\bar{f}\left(\theta^{*},a\right)-\bar{f}\left(a\right)\right)}^{2}|{\hat{a}}_{t}=a^{\prime }\right]\\ \; & \geq \sum_{a,a^{\prime }}{\bar{P}}_{t}\left(a_{t}=a\right){\bar{P}}_{t}\left({\hat{a}}_{t}=a^{\prime }\right){\left({\bar{E}}_{t}\left[\left(\bar{f}\left(\theta^{*},a\right)-\bar{f}\left(a\right)\right)|{\hat{a}}_{t}=a^{\prime }\right]\right)}^{2} \end{array}$$

(A83) $$\begin{array}{cc} \; & =\sum_{a,a^{\prime }}{\bar{P}}_{t}\left(a_{t}=a\right){\bar{P}}_{t}\left({\hat{a}}_{t}=a^{\prime }\right){\left({\bar{E}}_{t}[\left(\bar{f}\left(\theta^{*},a\right)|{\hat{a}}_{t}=a^{\prime }\right]-\bar{f}\left(a\right)\right)}^{2}={∥M∥}_{F}^{2}, \end{array}$$

whereby we obtain

(A84) $$\begin{array}{c} \Gamma_{t}\leq \frac{\mathrm{Tr}{\left(M\right)}^{2}}{{∥M∥}_{F}^{2}}\leq m, \end{array}$$

where the last inequality can be proved as in [25]. Following [19], it can be seen that $\Lambda_{t}\leq 2\log\left(1+K\right)$ also holds. Using this, together with the upper bound (A79) gives

(A85) $$\begin{array}{c} \sum_{t=1}^{T}E\left[{\mathrm{Term}}_{1}\right]\leq \sqrt{2Tm\sigma^{2}\min\left\{m,2\log\left(1+K\right)\right\}\log\left(1+\frac{T\lambda }{m\sigma^{2}}\right)}. \end{array}$$

### Appendix C.3. Proof of Lemma 2

We first give an upper bound on the estimation error that does not require the assumption of a linear feature map.

#### Appendix C.3.1. A General Upper Bound on $R_{\mathrm{EE}1}^{T}$

To obtain an upper bound on $R_{\mathrm{EE}1}^{T}$ that does not require the assumption that ϕ(a,c)=G(a)c, we leverage the same analysis as in the proof of Lemma 4. Subsequently, we obtain that

$$\begin{array}{cc} R_{\mathrm{EE}1}^{T} & \leq \sum_{t=1}^{T}E\left[\Delta \left(P\left(c_{t}|{\hat{c}}_{t},\gamma^{*}\right),P\left(c_{t}|{\hat{c}}_{t},H_{\hat{c}}\right)\right)1\left\{E\right\}\right]+2\delta TE[∥\theta^{*}{∥}_{2}\left|E^{c}\right]\\ \; & \leq \sum_{t=1}^{T}E\left[\Delta \left(P\left(c_{t}|{\hat{c}}_{t},\gamma^{*}\right),P\left(c_{t}|{\hat{c}}_{t},H_{\hat{c}}\right)\right)1\left\{E\right\}\right]+2\delta^{2}T\sqrt{\frac{m\lambda }{2\pi }}, \end{array}$$

where the event E is defined as in (A37). Subsequently, the first summation can be upper bounded as

(A86) $$\begin{array}{cc} \sum_{t=1}^{T}E[\Delta \left(P\left(c_{t}|{\hat{c}}_{t},\gamma^{*}\right),P\left(c_{t}|{\hat{c}}_{t},H_{\hat{c}}\right)\right)1\left\{E\right\}] & \leq \sqrt{2TU^{2}\sum_{t=1}^{T}I\left(c_{t};\gamma^{*}|{\hat{c}}_{t},H_{\hat{c}}\right)}\\ \; & =\sqrt{2TU^{2}\sum_{t=1}^{T}\left(H\left(c_{t}|{\hat{c}}_{t},H_{\hat{c}}\right)-H\left(c_{t}|{\hat{c}}_{t},\gamma^{*}\right)\right)}\\ \; & =\sqrt{TU^{2}\sum_{t=1}^{T}\log\left(\det\left(R_{t}^{-1}\right)\det\left(M_{t}\right)\right)}\\ \; & \leq \sqrt{TU^{2}\left(\mathrm{Tr}\left({\left(\Sigma_{n}\Sigma_{\gamma }^{-1}\Sigma_{n}M\right)}^{-1}\right)+\log\left(T\right)\mathrm{Tr}\left(\Sigma_{c}\Sigma_{n}^{-1}\right)\right)} \end{array}$$

where *U* is defined as in (A37), $M_{t}=\Sigma_{c}^{-1}+\Sigma_{n}^{-1}$ and $R_{t}$ is as in (13).

To derive the last inequality, we observe the following series of relationships starting from (13):

$$\begin{array}{cc} \Sigma_{n}^{-1}{\left(H_{t}^{-1}\right)}^{⊤}\Sigma_{n}^{-1} & ={\left(\Sigma_{n}H_{t}\Sigma_{n}\right)}^{-1}={\left(\left(t-1\right)\Sigma_{n}-\left(t-2\right)M^{-1}+\Sigma_{n}\Sigma_{\gamma }^{-1}\Sigma_{n}\right)}^{-1}\\ R_{t} & =M-\Sigma_{n}^{-1}{\left(H_{t}^{-1}\right)}^{⊤}\Sigma_{n}^{-1}\\ \; & =M-{\left(\left(t-1\right)\Sigma_{n}-\left(t-2\right)M^{-1}+\Sigma_{n}\Sigma_{\gamma }^{-1}\Sigma_{n}\right)}^{-1}\\ \; & =M\left[I-M^{-1}{\left(\left(t-1\right)\Sigma_{n}-\left(t-2\right)M^{-1}+\Sigma_{n}\Sigma_{\gamma }^{-1}\Sigma_{n}\right)}^{-1}\right]\\ R_{t}^{-1} & ={\left[I-M^{-1}{\left(\left(t-1\right)\Sigma_{n}-\left(t-2\right)M^{-1}+\Sigma_{n}\Sigma_{\gamma }^{-1}\Sigma_{n}\right)}^{-1}\right]}^{-1}M^{-1}\\ \; & ={\left[I-{\left(\left(t-1\right)\Sigma_{n}M-\left(t-2\right)I+\Sigma_{n}\Sigma_{\gamma }^{-1}\Sigma_{n}M\right)}^{-1}\right]}^{-1}M^{-1} \end{array}$$

$$\begin{array}{cc} R_{t}^{-1}M & ={\left[I-{\left(\left(t-1\right)\Sigma_{n}M-\left(t-2\right)I+\Sigma_{n}\Sigma_{\gamma }^{-1}\Sigma_{n}M\right)}^{-1}\right]}^{-1}\\ \; & ={\left[I-{\left(\left(t-1\right)\Sigma_{n}\Sigma_{c}^{-1}+\left(t-1\right)I-\left(t-2\right)I+\Sigma_{n}\Sigma_{\gamma }^{-1}\Sigma_{n}M\right)}^{-1}\right]}^{-1}\\ \; & ={\left[I-{\left(I+\underset{:=P_{t}}{\underset{︸}{\left(t-1\right)\Sigma_{n}\Sigma_{c}^{-1}+\Sigma_{n}\Sigma_{\gamma }^{-1}\Sigma_{n}M}}\right)}^{-1}\right]}^{-1}\\ \; & \overset{\left(a\right)}{=}{\left[I-\left(I-P_{t}{\left(I+P_{t}\right)}^{-1}\right)\right]}^{-1}={\left(P_{t}{\left(I+P_{t}\right)}^{-1}\right)}^{-1} \end{array}$$

where the equality in (a) follows from Woodbury matrix identity and by the assumption that $\Sigma_{n}\Sigma_{\gamma }^{-1}\Sigma_{n}M≻0$, $\Sigma_{n}\Sigma_{c}^{-1}≻0$, we have $P_{t}≻0$ is invertible. Now,

$$\begin{array}{cc} \det(R_{t}^{-1}M) & =\frac{1}{\det(P_{t}{\left(I+P_{t}\right)}^{-1})}=\det\left(P_{t}^{-1}\left(I+P_{t}\right)\right)\\ & =\det(P_{t}^{-1}+I)\\ \; & \overset{\left(b\right)}{\leq }{\left(\frac{\mathrm{Tr}(P_{t}^{-1}+I)}{d}\right)}^{d}={\left(1+\mathrm{Tr}\left(P_{t}^{-1}\right)/d\right)}^{d}, \end{array}$$

where the inequality in (b) follows from the determinant-trace inequality. Subsequently, we have

(A87) $$\begin{array}{cc} \sum_{t=1}^{T}\log\left(\det(R_{t}^{-1}M)\right) & \leq \sum_{t=1}^{T}d\log\left(1+\mathrm{Tr}\left(P_{t}^{-1}\right)/d\right)\\ \; & \overset{\left(c\right)}{\leq }\sum_{t=1}^{T}\mathrm{Tr}\left(P_{t}^{-1}\right)\\ \; & =\mathrm{Tr}\left(P_{1}^{-1}\right)+\sum_{t=2}^{T}\mathrm{Tr}\left(P_{t}^{-1}\right)\\ \; & =\mathrm{Tr}\left({\left(\Sigma_{n}\Sigma_{\gamma }^{-1}\Sigma_{n}M\right)}^{-1}\right)+\sum_{t=2}^{T}\mathrm{Tr}\left(P_{t}^{-1}\right)\\ \; & \overset{\left(d\right)}{\leq }\mathrm{Tr}\left({\left(\Sigma_{n}\Sigma_{\gamma }^{-1}\Sigma_{n}M\right)}^{-1}\right)+\log\left(T\right)\mathrm{Tr}\left(\Sigma_{c}\Sigma_{n}^{-1}\right), \end{array}$$

where the inequality in (c) follows since log(1+x)≤x for x>0 and the inequality in (d) follows since by assumption $P_{t}⪰\left(t-1\right)\Sigma_{n}\Sigma_{c}^{-1}$, whereby we have $P_{t}^{-1}⪯\frac{1}{t-1}\Sigma_{c}\Sigma_{n}^{-1}$ and consequently, $\mathrm{Tr}\left(P_{t}^{-1}\right)\leq \frac{1}{t-1}\mathrm{Tr}\left(\Sigma_{c}\Sigma_{n}^{-1}\right)$. Finally, we use that $\sum_{s=1}^{T}1/s\leq \log\left(T\right)$.

We thus obtain that

(A88) $$\begin{array}{c} R_{\mathrm{EE}1}^{T}\leq \sqrt{2\lambda mT\log\left(2m/\delta \right)\left(\mathrm{Tr}\left({\left(\Sigma_{n}\Sigma_{\gamma }^{-1}\Sigma_{n}M\right)}^{-1}\right)+\log\left(T\right)\mathrm{Tr}\left(\Sigma_{c}\Sigma_{n}^{-1}\right)\right)}+2\delta^{2}T\sqrt{\frac{m\lambda }{2\pi }} \end{array}$$

for δ∈(0,1). We note that same upper bound holds for the term $R_{\mathrm{EE}2}^{T}$.

#### Appendix C.3.2. Upper Bound for Linear Feature Maps and Scaled Diagonal Covariance Matrices

We now obtain an upper bound on the estimation error under the assumption of a linear feature map $\phi \left(a,c\right)=G_{a}c$ such that ${∥\phi \left(a,c\right)∥}_{2}\leq 1$. The following set of inequalities hold:

(A89) $$\begin{array}{cc} R_{\mathrm{EE}1}^{T} & =\sum_{t=1}^{T}E\left[\psi {\left(a_{t}^{*},{\hat{c}}_{t}|\gamma^{*}\right)}^{⊤}\theta^{*}-\psi {\left({\hat{a}}_{t},{\hat{c}}_{t}|H_{\hat{c}}\right)}^{⊤}\theta^{*}\right]\\ & \leq \sum_{t=1}^{T}E\left[\psi {\left(a_{t}^{*},{\hat{c}}_{t}|\gamma^{*}\right)}^{⊤}\theta^{*}-\psi {\left(a_{t}^{*},{\hat{c}}_{t}|H_{\hat{c}}\right)}^{⊤}\theta^{*}\right]\\ & =\sum_{t=1}^{T}E[E_{P(c_{t}|{\hat{c}}_{t},\gamma^{*})}\left[\phi {\left(a_{t}^{*},c_{t}\right)}^{⊤}\theta^{*}\right]-E_{P(c_{t}|{\hat{c}}_{t},H_{\hat{c}})}\left[\phi {\left(a_{t}^{*},c_{t}\right)}^{⊤}\theta^{*}\right]. \end{array}$$

Note, that $P\left(c_{t}|{\hat{c}}_{t},H_{\hat{c}}\right)=N\left(c_{t}|V_{t},R_{t}^{-1}\right)$ where $R_{t}$ and $V_{t}$ are, respectively, defined in (13) and (14). Consequently, $\phi {\left(a_{t}^{*},c_{t}\right)}^{⊤}\theta^{*}=c_{t}^{⊤}G_{a_{t}^{*}}^{⊤}\theta^{*}$ is $s_{t}^{2}=\theta^{*⊤}G_{a_{t}^{*}}R_{t}^{-1}G_{a_{t}^{*}}^{⊤}\theta^{*}$-sub-Gaussian with respect to $P(c_{t}|{\hat{c}}_{t},H_{\hat{c}})$. Consequently, using Lemma A1, we can upper bound the inner expectation of (A89) as

(A90) $$\begin{array}{c} |E_{P(c_{t}|{\hat{c}}_{t},\gamma^{*})}\left[\phi {\left(a_{t}^{*},c_{t}\right)}^{⊤}\theta^{*}\right]-E_{P(c_{t}|{\hat{c}}_{t},H_{\hat{c}})}\left[\phi {\left(a_{t}^{*},c_{t}\right)}^{⊤}\theta^{*}\right|\leq \sqrt{2s_{t}^{2}D_{\mathrm{KL}}\left(P\left(c_{t}|{\hat{c}}_{t},\gamma^{*}\right)∥P\left(c_{t}|{\hat{c}}_{t},H_{\hat{c}}\right)\right)}. \end{array}$$

Summing over *t* and using Cauchy–Schwarz inequality then gives

(A91) $$\begin{array}{cc} R_{\mathrm{EE}1}^{T} & \leq \sqrt{2\left(\sum_{t=1}^{T}E\left[s_{t}^{2}\right]\right)\left(\sum_{t=1}^{T}E\left[D_{\mathrm{KL}}\left(P\left(c_{t}|{\hat{c}}_{t},\gamma^{*}\right)∥P\left(c_{t}|{\hat{c}}_{t},H_{\hat{c}}\right)\right)\right]\right)}. \end{array}$$

We now evaluate the KL-divergence term. To this end, note that conditioned on $\gamma^{*}$ and ${\hat{c}}_{t}$, $c_{t}$ is independent of $H_{\hat{c}}$, i.e., $P\left(c_{t}|{\hat{c}}_{t},\gamma^{*},H_{\hat{c}}\right)=P\left(c_{t}|{\hat{c}}_{t},\gamma^{*}\right)$. This gives

(A92) $$\begin{array}{c} \sum_{t=1}^{T}E\left[D_{\mathrm{KL}}\left(P\left(c_{t}|{\hat{c}}_{t},\gamma^{*}\right)∥P\left(c_{t}|{\hat{c}}_{t},H_{\hat{c}}\right)\right)\right]\\ =\sum_{t=1}^{T}I\left(c_{t};\gamma^{*}|{\hat{c}}_{t},H_{\hat{c}}\right)\\ =\sum_{t=1}^{T}H\left(c_{t}|{\hat{c}}_{t},H_{\hat{c}}\right)-H\left(c_{t}|{\hat{c}}_{t},\gamma^{*}\right)\\ =\frac{1}{2}\sum_{t=1}^{T}E_{P({\hat{c}}_{t},H_{\hat{c}})}\left[\log\det\left(R_{t}^{-1}\right)\right]-\frac{1}{2}\sum_{t=1}^{T}E_{P({\hat{c}}_{t},\gamma^{*})}\left[\log\det\left(M^{-1}\right)\right]\\ =\sum_{t=1}^{T}\frac{1}{2}E_{P({\hat{c}}_{t},H_{\hat{c}})}[\log\left(\det\left(R_{t}^{-1}\right)\det\left(M\right)\right)\\ \leq \frac{d\sigma_{c}^{2}}{\sigma_{n}^{2}}\left(\frac{\sigma_{\gamma }^{2}}{\sigma_{n}^{2}+\sigma_{c}^{2}}+\log\left(T-1\right)\right), \end{array}$$

where $M=\Sigma_{c}^{-1}+\Sigma_{n}^{-1}$ and $R_{t}$ is as in (13). The first equality follows by noting that

$$\begin{array}{cc} E\left[D_{\mathrm{KL}}\left(P\left(c_{t}|{\hat{c}}_{t},\gamma^{*}\right)∥P\left(c_{t}|{\hat{c}}_{t},H_{\hat{c}}\right)\right)\right] & =E_{P({\hat{c}}_{t},H_{\hat{c}})}\left[E_{P(\gamma^{*}|{\hat{c}}_{t},H_{\hat{c}})}\left[D_{\mathrm{KL}}\left(P\left(c_{t}|{\hat{c}}_{t},\gamma^{*},H_{\hat{c}}\right)∥P\left(c_{t}|{\hat{c}}_{t},H_{\hat{c}}\right)\right)\right]\right]\\ & =E_{P({\hat{c}}_{t},H_{\hat{c}})}\left[I\left(c_{t};\gamma^{*}|{\hat{c}}_{t},H_{\hat{c}}\right)\right] \end{array}$$

with the outer expectation taken over ${\hat{c}}_{t}$ and $H_{\hat{c}}$. The last inequality is proved in Appendix C.3.3 using that $\Sigma_{c}=\sigma_{c}^{2}I$, $\Sigma_{n}=\sigma_{n}^{2}I$ and $\Sigma_{\gamma }=\sigma_{\gamma }^{2}I$.

We can now upper bound $\sum_{t}E\left[s_{t}^{2}\right]$ as follows.

$$\begin{array}{cc} E\left[s_{t}^{2}\right]\leq E\left[\max_{a}\theta^{*⊤}G_{a}R_{t}^{-1}G_{a}^{⊤}\theta^{*}\right]\leq \sum_{a}E\left[\theta^{*⊤}G_{a}R_{t}^{-1}G_{a}^{⊤}\theta^{*}\right] & =\sum_{a}\mathrm{Tr}\left(G_{a}R_{t}^{-1}G_{a}^{⊤}E\left[\theta^{*}\theta^{*⊤}\right]\right)\\ & =\lambda \sum_{a}\mathrm{Tr}\left(R_{t}^{-1}G_{a}^{⊤}G_{a}\right)\\ \; & =\lambda b_{t}\mathrm{Tr}\left(\sum_{a}G_{a}^{⊤}G_{a}\right) \end{array}$$

where the last equality uses $R_{t}^{-1}=b_{t}I$ as in (A95). Using (A95), we obtain

$$\begin{array}{cc} \sum_{t}b_{t} & =\frac{\sigma_{c}^{2}\sigma_{n}^{2}}{f}\sum_{t}\left(1+\frac{\sigma_{c}^{2}\sigma_{\gamma }^{2}}{\left(t-1\right)\sigma_{\gamma }^{2}\sigma_{n}^{2}+f\sigma_{n}^{2}}\right)\\ \; & =\frac{\sigma_{c}^{2}\sigma_{n}^{2}T}{f}+\sum_{t}\frac{\sigma_{c}^{2}}{f}\frac{\sigma_{c}^{2}\sigma_{\gamma }^{2}}{\left(t-1\right)\sigma_{\gamma }^{2}+f}\\ \; & =\frac{\sigma_{c}^{2}\sigma_{n}^{2}T}{f}+\frac{\sigma_{c}^{4}\sigma_{\gamma }^{2}}{f^{2}}+\sum_{t>1}\frac{\sigma_{c}^{2}}{f}\frac{\sigma_{c}^{2}\sigma_{\gamma }^{2}}{\left(t-1\right)\sigma_{\gamma }^{2}+f}\\ \; & \leq \frac{\sigma_{c}^{2}\sigma_{n}^{2}}{f}T+\frac{\sigma_{c}^{4}\sigma_{\gamma }^{2}}{f^{2}}+\frac{\sigma_{c}^{4}}{f}\log\left(T-1\right). \end{array}$$

Using the above relation, we obtain

$$\begin{array}{c} \sum_{t}E\left[s_{t}^{2}\right]=\lambda \mathrm{Tr}\left(\sum_{a}G_{a}^{⊤}G_{a}\right)\sum_{t}b_{t}\leq \frac{\lambda K\sigma_{c}^{2}}{f}\max_{a}\mathrm{Tr}\left(G_{a}^{⊤}G_{a}\right)\left(\sigma_{n}^{2}T+\frac{\sigma_{c}^{2}\sigma_{\gamma }^{2}}{f}+\sigma_{c}^{2}\log\left(T-1\right)\right). \end{array}$$

If $\lambda \leq \frac{d\sigma^{2}}{T}$, we obtain

$$\begin{array}{c} \sum_{t}E\left[s_{t}^{2}\right]\leq \frac{d\sigma^{2}K\sigma_{c}^{2}}{f}\max_{a}\mathrm{Tr}\left(G_{a}^{⊤}G_{a}\right)\left(\sigma_{n}^{2}+\frac{\sigma_{c}^{2}\sigma_{\gamma }^{2}}{Tf}+\sigma_{c}^{2}\frac{\log(T-1)}{T}\right)=L \end{array}$$

Using the above inequality together with (A92) in (A91) yields

(A93) $$\begin{array}{c} R_{\mathrm{EE}1}^{T}\leq \sqrt{2L\frac{d\sigma_{c}^{2}}{\sigma_{n}^{2}}\left(\frac{\sigma_{\gamma }^{2}}{\sigma_{n}^{2}+\sigma_{c}^{2}}+\log\left(T-1\right)\right)}. \end{array}$$

An upper bound on $R_{\mathrm{EE}2}^{T}$ similarly follows.

#### Appendix C.3.3. Analysis of $R_{t}$ for Scaled Diagonal Covariance Matrices

Assume that $\Sigma_{n}=\sigma_{n}^{2}I$, $\Sigma_{c}=\sigma_{c}^{2}I$ and $\Sigma_{\gamma }=\sigma_{\gamma }^{2}I$. Then, from (13), we obtain

(A94) $$\begin{array}{cc} H_{t} & =\left(t-1\right)\Sigma_{n}^{-1}-\left(t-2\right)\Sigma_{n}^{-1}M^{-1}\Sigma_{n}^{-1}+\Sigma_{\gamma }^{-1}\\ \; & =(\frac{\left(t-1\right)}{\sigma_{n}^{2}}-\frac{\left(t-2\right)\sigma_{n}^{2}\sigma_{c}^{2}}{\sigma_{n}^{4}\underset{:=f}{\underset{︸}{\left(\sigma_{c}^{2}+\sigma_{n}^{2}\right)}}}+\frac{1}{\sigma_{\gamma }^{2}})I\\ \; & =\frac{\left(t-1\right)\sigma_{\gamma }^{2}-\left(t-2\right)\sigma_{c}^{2}\sigma_{\gamma }^{2}/f+\sigma_{n}^{2}}{\sigma_{n}^{2}\sigma_{\gamma }^{2}}I. \end{array}$$

This implies

$$\begin{array}{cc} H_{t}^{-1} & =\frac{\sigma_{n}^{2}\sigma_{\gamma }^{2}}{\left(t-1\right)\sigma_{\gamma }^{2}-\left(t-2\right)\sigma_{c}^{2}\sigma_{\gamma }^{2}/f+\sigma_{n}^{2}}I \end{array}$$

whereby we obtain

(A95) $$\begin{array}{cc} R_{t} & =\frac{f\left(\left(t-1\right)\sigma_{\gamma }^{2}+\sigma_{n}^{2}+\sigma_{c}^{2}\right)}{\sigma_{c}^{2}\left(\left(t-1\right)\sigma_{\gamma }^{2}\sigma_{n}^{2}+\sigma_{c}^{2}\sigma_{\gamma }^{2}+f\sigma_{n}^{2}\right)}I\;\mathrm{and}\\ R_{t}^{-1} & =\frac{\sigma_{c}^{2}\left(\left(t-1\right)\sigma_{\gamma }^{2}\sigma_{n}^{2}+\sigma_{c}^{2}\sigma_{\gamma }^{2}+f\sigma_{n}^{2}\right)}{f\left(\left(t-1\right)\sigma_{\gamma }^{2}+\sigma_{n}^{2}+\sigma_{c}^{2}\right)}I=b_{t}I. \end{array}$$

Noting that $M_{t}=\frac{f}{\sigma_{n}^{2}\sigma_{c}^{2}}I$ we then have

(A96) $$\begin{array}{cc} R_{t}^{-1}M_{t} & =\frac{\left(t-1\right)\sigma_{\gamma }^{2}\sigma_{n}^{2}+\sigma_{c}^{2}\sigma_{\gamma }^{2}+f\sigma_{n}^{2}}{\sigma_{n}^{2}\left(\left(t-1\right)\sigma_{\gamma }^{2}+\sigma_{n}^{2}+\sigma_{c}^{2}\right)}I=\frac{\left(t-1\right)\sigma_{\gamma }^{2}\sigma_{n}^{2}+\sigma_{c}^{2}\sigma_{\gamma }^{2}+f\sigma_{n}^{2}}{\left(t-1\right)\sigma_{\gamma }^{2}\sigma_{n}^{2}+f\sigma_{n}^{2}}I=\left(1+\frac{\sigma_{c}^{2}\sigma_{\gamma }^{2}}{\left(t-1\right)\sigma_{\gamma }^{2}\sigma_{n}^{2}+f\sigma_{n}^{2}}\right)I. \end{array}$$

Subsequently, we obtain that for t>1,

(A97) $$\begin{array}{c} \log\left(\det(R_{t}^{-1}M_{t})\right)=d\log\left(1+\frac{\sigma_{c}^{2}\sigma_{\gamma }^{2}}{\left(t-1\right)\sigma_{\gamma }^{2}\sigma_{n}^{2}+f\sigma_{n}^{2}}\right)\leq d\log\left(1+\frac{\sigma_{c}^{2}/\sigma_{n}^{2}}{\left(t-1\right)}\right)\leq \frac{d\sigma_{c}^{2}/\sigma_{n}^{2}}{t-1}, \end{array}$$

whereby

(A98) $$\begin{array}{cc} \sum_{t=1}^{T}\log\left(\det(R_{t}^{-1}M_{t})\right) & \leq d\log\left(1+\frac{\sigma_{c}^{2}\sigma_{\gamma }^{2}}{f\sigma_{n}^{2}}\right)+\sum_{t>1}^{T}\frac{d\sigma_{c}^{2}/\sigma_{n}^{2}}{t-1} \end{array}$$

(A99) $$\begin{array}{cc} \; & \leq \frac{d\sigma_{c}^{2}}{\sigma_{n}^{2}}\left(\frac{\sigma_{\gamma }^{2}}{f}+\log\left(T-1\right)\right) \end{array}$$

where the last inequality follows since log(1+x)≤x and $\sum_{s=1}^{T}\frac{1}{s}\leq \log\left(T\right).$

## Appendix D. Details on Experiments

In this section, we present details on the baselines implemented for stochastic CBs with unobserved true contexts.

### Appendix D.1. Gaussian Bandits

For Gaussian bandits, we implemented the baselines as explained below.

*TS_naive:* This algorithm implements the following action policy at each iteration *t*,

$$\begin{array}{c} a_{t}=\arg\max_{a\in A}\phi {\left(a,{\hat{c}}_{t}\right)}^{⊤}\theta_{t}, \end{array}$$

where $\theta_{t}$ is sampled from a Gaussian distribution $N(\mu_{t-1,\mathrm{naive}},\Sigma_{t-1,\mathrm{naive}}^{-1})$ with

$$\begin{array}{cc} \Sigma_{t-1,\mathrm{naive}} & =\frac{I}{\lambda }+\frac{1}{\sigma^{2}}\sum_{\tau =1}^{t-1}\phi \left(a_{\tau },{\hat{c}}_{\tau }\right)\phi {\left(a_{\tau },{\hat{c}}_{\tau }\right)}^{⊤}\\ \mu_{t-1,\mathrm{naive}} & =\frac{\Sigma_{t-1,\mathrm{naive}}^{-1}}{\sigma^{2}}\left(\sum_{\tau =1}^{t-1}r_{\tau }\phi \left(a_{\tau },{\hat{c}}_{\tau }\right)\right). \end{array}$$

*TS_oracle:* In this baseline, the agent has knowledge of the true predictive distribution $P(c_{t}|{\hat{c}}_{t},\gamma^{*})$. Consequently, at each iteration *t*, the algorithm chooses action

$$\begin{array}{c} a_{t}=\arg\max_{a\in A}\psi {\left(a,{\hat{c}}_{t}|\gamma^{*}\right)}^{⊤}\theta_{t}, \end{array}$$

where $\theta_{t}$ is sampled from a Gaussian distribution $N(\mu_{t-1,\mathrm{poc}},\Sigma_{t-1,\mathrm{poc}}^{-1})$ with

$$\begin{array}{cc} \Sigma_{t-1,\mathrm{poc}} & =\frac{I}{\lambda }+\frac{1}{\sigma^{2}}\sum_{\tau =1}^{t-1}\psi \left(a_{\tau },{\hat{c}}_{\tau }|\gamma^{*}\right)\psi {\left(a_{\tau },{\hat{c}}_{\tau }|\gamma^{*}\right)}^{⊤}\\ \mu_{t-1,\mathrm{poc}} & =\frac{\Sigma_{t-1,\mathrm{poc}}^{-1}}{\sigma^{2}}\left(\sum_{\tau =1}^{t-1}r_{\tau }\psi \left(a_{\tau },{\hat{c}}_{\tau }|\gamma^{*}\right)\right). \end{array}$$

For Gaussian bandits, the following figure shows additional experiment comparing the performance of our proposed Algorithm 1 for varying values of the number *K* of actions. All parameters are set as in Figure 1 (Left).

### Appendix D.2. Logistic Bandits

In the case of logistic bandits, we implemented the baselines as explained below.

*TS_naive:* This algorithm considers the following sampling distribution:

$$\begin{array}{c} Q\left(\theta^{*}|H_{t-1,r,a,\hat{c}}\right)\propto P\left(\theta^{*}\right)\prod_{\tau =1}^{t-1}\mathrm{Ber}\left(\mu \left(\phi {\left(a_{\tau },{\hat{c}}_{\tau }\right)}^{⊤}\theta^{*}\right)\right). \end{array}$$

However, due to the non-conjugateness of Gaussian prior $P(\theta^{*})$ and Bernoulli reward likelihood, sampling from the above posterior distribution is not straightforward. Consequently, we adopt the Langevin Monte Carlo (LMC) sampling approach from [20]. To sample $\theta_{t}$ at iteration *t*, we run LMC for I=50 iterations with learning rate $\eta_{t}=0.2/t$ and inverse temperature parameter $\beta^{-1}=0.001$. Then, $\theta_{t}$ is chosen as the output of the LMC after I=50 iterations. Using the sampled $\theta_{t}$, the algorithm then chooses the action $a_{t}$ as

$$\begin{array}{c} a_{t}=\arg\max_{a\in A}\phi {\left(a,{\hat{c}}_{t}\right)}^{⊤}\theta_{t}. \end{array}$$

*TS_oracle:* This algorithm considers the following sampling distribution:

$$\begin{array}{c} Q\left(\theta^{*}|H_{t-1,r,a,\hat{c}}\right)\propto P\left(\theta^{*}\right)\prod_{\tau =1}^{t-1}\mathrm{Ber}\left(\mu \left(\psi {\left(a_{\tau },{\hat{c}}_{\tau }|\gamma^{*}\right)}^{⊤}\theta^{*}\right)\right), \end{array}$$

where $\psi \left(a_{t},{\hat{c}}_{t}|\gamma^{*}\right)=E_{P(c_{t}|{\hat{c}}_{t},\gamma^{*})}\left[\phi \left(a_{t},c_{t}\right)\right]$ is the expected feature map under the posterior predictive distribution with known $\gamma^{*}$. As before, to sample from the above distribution, we use I=50 iterations of LMC with learning rate $\eta_{t}=0.2/t$ and inverse temperature parameter $\beta^{-1}=0.001$. Using the sampled $\theta_{t}$, the algorithm then chooses the action $a_{t}$ as

$$\begin{array}{c} a_{t}=\arg\max_{a\in A}\psi {\left(a,{\hat{c}}_{t}|\gamma^{*}\right)}^{⊤}\theta_{t}. \end{array}$$
